# High-accuracy laser spectroscopy of ${{\bf{H}}}_{{\bf{2}}}^{{\boldsymbol{+}}}$ and the proton–electron mass ratio

**DOI:** 10.1038/s41586-025-09306-2

**Published:** 2025-08-06

**Authors:** S. Alighanbari, M. R. Schenkel, V. I. Korobov, S. Schiller

**Affiliations:** 1 Heinrich-Heine-Universität Düsseldorf, Mathematisch-Naturwissenschaftliche Fakultät, Institut für Experimentalphysik, Düsseldorf, Germany; 2 Bogoliubov Laboratory of Theoretical Physics, Joint Institute for Nuclear Research, Dubna, Russia

**Keywords:** Atomic and molecular interactions with photons, Optical metrology, Quantum metrology

## Abstract

The molecular hydrogen ions (MHI) are three-body systems suitable for advancing our knowledge in several domains: fundamental constants, tests of quantum physics, search for new interparticle forces, tests of the weak equivalence principle^1^ and, once the anti-molecule $\overline{p}\,\overline{p}\,{e}^{+}$ becomes available, new tests of charge–parity–time-reversal invariance and local position invariance^1–3^. To achieve these goals, high-accuracy laser spectroscopy of several isotopologues, in particular ${{\rm{H}}}_{2}^{+}$, is required^4^. Here we present a Doppler-free laser spectroscopy of a ${{\rm{H}}}_{2}^{+}$ rovibrational transition, achieving line resolutions as large as 2.2 × 10^13^. We accurately determine the transition frequency with 8 × 10^−12^ fractional uncertainty. We also determine the spin–rotation coupling coefficient with 0.1 kHz uncertainty and its value is consistent with the state-of-the-art theory prediction^5^. The combination of our theoretical and experimental ${{\rm{H}}}_{2}^{+}$ data allows us to deduce a new value for the proton-electron mass ratio *m*_p_/*m*_e_. It is in agreement with the value obtained from mass spectrometry and has 2.3 times lower uncertainty. From combined MHI, H/D and muonic H/D data, we determine the baryon mass ratio *m*_d_/*m*_p_ with 1.1 × 10^−10^ absolute uncertainty. The value agrees with the directly measured mass ratio^6^. Finally, we present a match between a theoretical prediction and an experimental result, with a fractional uncertainty of 8.1 × 10^−12^. Both results indicate a notable confirmation of the predictive power of quantum theory and the absence of beyond-the-standard-model effects at these levels.

## Abstract (web summary)

Doppler-free laser spectroscopy is used to measure with high precision a rovibrational transition frequency of trapped and cooled ${{\rm{H}}}_{2}^{+}$ ions.

## Main

The relative simplicity of the molecular hydrogen ions (MHI) allows for the accurate computation of their properties. At present, the fractional uncertainty of the predictions of rovibrational transition frequencies is 8 × 10^−12^ (ref. ^7^), only a factor of approximately 10 larger than for the well-known 1*s*–2*s* transition of the theoretically more easily tractable hydrogen atom^8^. The calculations can be performed with equal accuracy for any member of the MHI family. Crucially, the calculations require several fundamental constants as input: the Rydberg constant *R*_*∞*_, the ratio *m*_e_/*m*_n_ of electron mass to the mass of any present nucleus n, the charge radius *r*_n_ of any such nucleus and the fine structure constant *α*. These constants are required for basic reasons: *cR*_*∞*_ defines the energy scale of all atomic and molecular binding energies and the mass ratios determine the rotational and vibrational energies relative to the energy scale. The nuclear charge radii subtly affect the potential experienced by the electron. Finally, *α* determines the strength of relativistic and quantum electrodynamics (QED) contributions to the energy of the electron. The impacts of these effects depend on internuclear distance and, therefore, are also discernible in vibrational transition frequencies. Except for *α*, the uncertainties *u* of the constants are not negligible when comparing the most precisely computed and measured frequencies. Therefore, a high-accuracy measurement of any transition, rotational or rovibrational, of any MHI species can contribute to reducing the uncertainty of those constants. Importantly, from MHI spectroscopy data and theory, combined with spectroscopy data and theory on atomic hydrogen, atomic deuterium, muonic hydrogen (µH) and muonic deuterium (μD), it is possible to determine the proton–electron *m*_p_/*m*_e_ and deuteron–proton *m*_d_/*m*_p_ mass ratios (refs. ^4,9,10^). This approach is independent and complementary to the more established approach based on mass spectrometry^6^ and electron spin resonance (ESR) in Penning traps^11^. This situation is highly beneficial to the field of fundamental constants, as measurements of the same quantities are made by different teams and involve different theoretical calculations. However, the same theoretical framework (QED) is used, specifically, its nonrelativistic limit^12^. Until now, measurements of rotational and rovibrational transitions of MHI with competitive accuracy have been accomplished only in the heteronuclear HD^+^ (refs. ^13–16^).

Table 1 presents an overview of selected approaches for determining the mass ratios of electron, proton and deuteron, and *r*_p_ and *r*_d_. Although these constants can be determined with well-known approaches (data rows 1, 4, 6), MHI results can, in principle, provide independent values of the constants or contribute data for a potentially more precise determination based on the complete dataset. For example, in the second data row, HD^+^ rovibrational data and its theory, combined with *r*_p_ from μH spectroscopy, *R*_*∞*_ from atomic hydrogen spectroscopy and *r*_d_ from the H/D isotope shift (and corresponding theory), yield the ratio of reduced nuclear mass, ${\mu }_{{\rm{pd}}}=1/({m}_{{\rm{p}}}^{-1}+{m}_{{\rm{d}}}^{-1})$, to electron mass *m*_e_ (ref. ^16^). This ratio result has furthermore been combined with a very accurate measurement of *m*_p_/*m*_d_, obtained using a Penning trap storing alternatively a deuteron and a ${{\rm{H}}}_{2}^{+}$ ion^6^, to provide the proton–electron mass ratio ${({m}_{{\rm{p}}}/{m}_{{\rm{e}}})}_{{{\rm{HD}}}^{+}}$. It can be compared with an independent determination from ESR on a single hydrogen-like ion^11^ (first data row). The two values are in agreement.

**Table 1:** Overview of some approaches suitable for the determination of fundamental constants with high accuracy

Main constants to be determined	Method and system							Reference
	Classical		Quantum					
	Cyclotron motion		Electron spin resonance, QED theory	Laser spectroscopy, QED theory				
	p, ion	${{\bf{H}}}_{{\bf{2}}}^{{\boldsymbol{+}}}$, d	Hydrogen-like ion	H, D	μH, μD	HD^+^	${{\bf{H}}}_{{\bf{2}}}^{{\boldsymbol{+}}}$	
*m*_p_/*m*_e_	**✓**		**✓**					CODATA 2018
		**✓**		**✓**	**✓**	**✓**		Reference ^16^ and CODATA 2018
				**✓**	**✓**		**✓**	LSA1: this work and CODATA 2018
*m*_d_/*m*_p_		**✓**						Reference ^6^
				**✓**	**✓**	**✓**	**✓**	LSA2: this work and CODATA 2018
*r*_p_, *r*_d_				**✓**	**✓**			CODATA 2018
		**✓**		**✓**		**✓**	**✓**	LSA3: this work and CODATA 2018
*r*_p_, *r*_d_, *m*_p_/*m*_e_		**✓**		**✓**	**✓**	**✓**	**✓**	LSA4: this work and CODATA 2018
								

Check marks indicate a measurement required to obtain the constant(s) in the first column of the same row. The bold check marks indicate the contributions of the present work (see text for details). Note that the table is not comprehensive; for example, the charge radii *r*_p_ and *r*_d_ can also be determined by electron scattering. The bottom row shows the schematic depictions of the systems used for the determinations. Orange ball, atomic ion; red ball, proton; and blue ball, deuteron. CODATA, Committee on Data of the International Science Council.

New possibilities arise if high-accuracy data for ${{\rm{H}}}_{2}^{+}$ become available (Table 1, third, fifth, seventh and eighth data rows). However, ${{\rm{H}}}_{2}^{+}$ experiments are challenging. In the past, determinations of rotational or vibrational transition frequencies have been limited to uncertainties above 1 × 10^−6^ (refs. ^17–19^), much larger than the current theoretical uncertainty, 8 × 10^−12^. An experimental breakthrough was recently reported by us—laser spectroscopy of sympathetically cooled ${{\rm{H}}}_{2}^{+}$ by an electric quadrupole (E2) transition^20^, reaching 1.2 × 10^−8^ uncertainty. Studies of Rydberg states of neutral H_2_ (refs. ^21,22^) can also provide data on ${{\rm{H}}}_{2}^{+}$, and a recent experiment determined a vibrational frequency with 8 × 10^−9^ uncertainty^23^.

In the present work, we improve direct ${{\rm{H}}}_{2}^{+}$ spectroscopy by three orders of magnitude in accuracy and succeed in matching the theoretical prediction uncertainty for a ${{\rm{H}}}_{2}^{+}$ vibrational transition. Our measurement and the corresponding theoretical calculation jointly provide a milestone in the field of fundamental constants. We obtain a new, purely laser-spectroscopic value of *m*_p_/*m*_e_ and, together with our previous HD^+^ data, a purely spectroscopic value of *m*_d_/*m*_p_. In both cases, atomic laser spectroscopy data contribute. These two values can be compared with those obtained from the respective Penning trap experiments^6,24^. Further combinations of measurement results are also considered.

## Laser spectroscopy of a vibrational transition in ${{\bf{H}}}_{{\bf{2}}}^{{\boldsymbol{+}}}$

A suitable method to accomplish vibrational spectroscopy of ${{\rm{H}}}_{2}^{+}$ is E2 spectroscopy^25^. As this is a type of one-photon spectroscopy, strong confinement of the molecules in the direction along the propagation of the spectroscopy laser is necessary to observe Doppler-free lines^15,16,20^. For this purpose, our experiment uses a linear radiofrequency (RF) ion trap to confine a small number of MHI, which are then sympathetically cooled to millikelvin temperature by interactions with a cluster of laser-cooled beryllium ions^26^. This results in the MHI being confined close to the symmetry axis of the trap. The spectroscopy beam is aligned perpendicular to the trap axis. Previously, we demonstrated the feasibility of Doppler-free E2 spectroscopy, using the heteronuclear HD^+^ for ease of experimentation^20^. A suitable continuous-wave laser system, with sufficient power and narrow linewidth, is a key instrument for this spectroscopy^27^. Further details on the experimental technique are provided in the Methods.

**Fig. 1:**
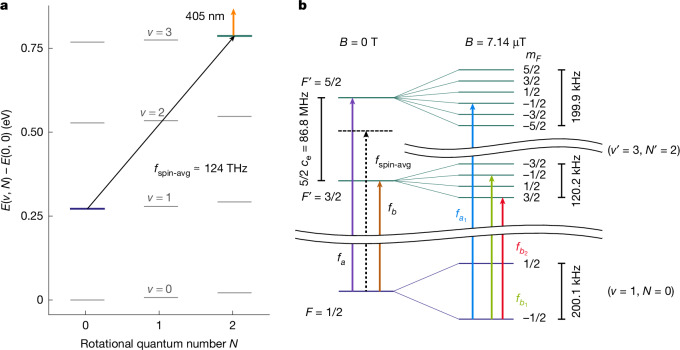
Energy levels of ${{\bf{H}}}_{{\bf{2}}}^{{\boldsymbol{+}}}$ and transitions relevant to this work. **a**, The lowest three rotational levels of the vibrational states *v* = 0–3. The studied transition (*v* = 1, *N* = 0) → (*v*′ = 3, *N*′ = 2) is shown by the black arrow, whereas the dissociation radiation is indicated by the orange arrow. **b**, Spin (left) and Zeeman structure (right) of the two rovibrational levels addressed in the present study. The upper vibrational level *v*′ = 3 consists of two states *F*′ = 3/2, 5/2 that are split by 86.8 MHz by the interaction between rotation and the magnetic moment of the electron. The two unperturbed spin components *f*_*a*_ and *f*_*b*_ of the transition are shown in purple and brown, respectively. The spin-averaged transition frequency *f*_spin-avg_ is not directly measured, but is indicated schematically as a black-dashed arrow. On the right side, the Zeeman splittings are shown for the nominal field applied during spectroscopy, *B*_REMPD_ = 7.14 μT. To show the Zeeman splitting, the vertical axis is broken at two positions. On the far right, the three coloured arrows (blue, green and red) indicate the measured Zeeman components ${f}_{{a}_{1}}$, ${f}_{{b}_{1}}$ and ${f}_{{b}_{2}}$. *F* and *F*′ are the total angular momentum of the molecule, *m*_*F*_ is the total angular momentum projection quantum number.

It is advantageous to select a transition whose spin structure is simple, as this allows the spin-averaged transition frequency to be obtained by effectively cancelling the spin structure^28^. The transition should also yield a sufficient signal to enable the measurement of systematic effects to a desired level of accuracy. We found that these conditions are met for the (*v* = 1, *N* = 0) → (*v*′ = 3, *N*′ = 2) vibrational transition at a wavelength of 2.4 μm (124 THz), which we have previously studied in the Doppler-broadened regime. Here, *v* and *N* denote vibrational and rotational quantum numbers, respectively. Figure 1 shows the lowest rotational and rovibrational levels of ${{\rm{H}}}_{2}^{+}$ and the spin structure of the energy levels. The latter is simple because for zero or even *N* (so-called para-${{\rm{H}}}_{2}^{+}$), the two proton spins must be in a singlet total spin state, *I* = 0. This leaves as the only remaining spin interaction the electron–spin–rotation interaction, *hc*_e_(*v*, *N*)**S**_**e**_ ⋅ **N**. Here, *c*_e_ is the coupling coefficient, **S**_**e**_ is the electron spin operator, **N** is the rotational angular momentum operator, and *h* is the Planck constant. The possible spin energies are *hc*_e_(*v*, *N*)(*F*(*F* + 1) − *N*(*N* + 1) − 3/4)/2, where the total angular momentum may take on the values *F* = |*N* − 1/2| or *N* + 1/2. By the selection rules, the vibrational transition exhibits two spin components, namely, *f*_*a*_: (*F* = 1/2 → *F*′ = 5/2) and *f*_*b*_: (*F* = 1/2 → *F*′ = 3/2). The frequencies of the two components are separated by *f*_*a*_ − *f*_*b*_ = 5*c*_e_(*v*, *N*)/2. The spin-averaged transition frequency is *f*_spin-avg_ = (3*f*_*a*_ + 2*f*_*b*_)/5.

## Experiment

To observe Doppler-free lines in ${{\rm{H}}}_{2}^{+}$, we followed the protocol of our previous work on HD^+^. We achieve Doppler-free spectroscopy at the expense of substantially more effort than for HD^+^, because the preparation of a large fraction of molecules in the lower spectroscopy level was not feasible. One data point in ${{\rm{H}}}_{2}^{+}$ spectroscopy took about 5 h of experimentation, whereas it took approximately 40 min in our study of the fourth overtone transition of HD^+^ (ref. ^16^). The successful data collection leading to complete lines was only possible because of the excellent long-term stability of our laser metrology system and trap apparatus.

**Fig. 2:**
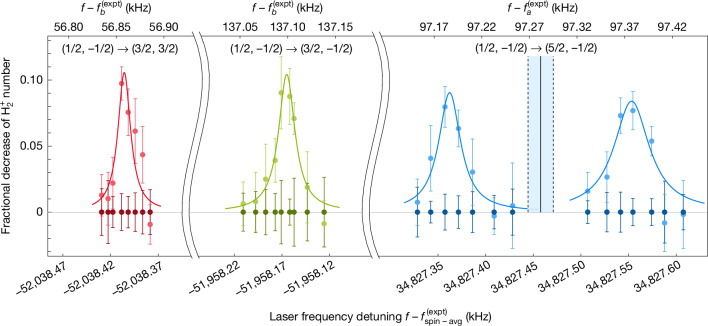
Zeeman components of the rovibrational transition measured under nominal operating conditions. The three Zeeman components ${f}_{{b}_{2}}$, ${f}_{{b}_{1}}$ and ${f}_{{a}_{1}}$ (from left to right) are shown with the same colours as in Fig. 1b. The labels in the panels indicate the quantum numbers, $(F,{m}_{F})\to ({F}^{{\prime} },{m}_{{F}^{{\prime} }})$. The component ${f}_{{a}_{1}}$ (light blue) is split into a doublet by the Autler–Townes (AT) effect (Methods). The deperturbed frequency and uncertainty range of this component are indicated by black full and dashed lines, respectively. We also assume that the Zeeman components of the other spin component (red and green) are AT-split, but did not measure the full AT doublets. The laser frequency detuning is given relative to different reference values in the top and the bottom abscissae. Top, the reference values are the deperturbed transition frequencies of the respective spin component ${f}_{a}^{({\rm{expt}})}$ and ${f}_{b}^{({\rm{e}}{\rm{x}}{\rm{p}}{\rm{t}})}$. Bottom, the reference value is the adjusted spin-averaged transition frequency ${f}_{\text{spin-avg}}^{({\rm{expt}})}$. The coloured curves are guides to the eye. For display purposes, the data are divided into bins, and the average value of each bin is shown. For an individual component, the bin size is kept constant, but it may vary between different components. The vertical error bars are the standard error of the mean in the bin. The horizontal error bars are due to the uncertainty of the frequency of the spectroscopy wave and are smaller than the size of a data point (Methods). Source Data

An excerpt of the recorded spectra is shown in Fig. 2. The narrowest linewidths observed were as low as around 6 Hz (Supplementary Fig. 4), corresponding to a line resolution of 2.2 × 10^13^. This represents the highest published resolution in molecular spectroscopy to date, to the best of our knowledge. It is higher by a factor of 7 compared with a cold-trapped-Sr_2_ spectroscopy experiment, in which a line resolution of 2.9 × 10^12^ was reported^29^. Furthermore, it is a factor of about 10^6^ higher than the only previous two studies of rovibrational spectroscopy of a homonuclear molecular ion transition^20,30^. The observed linewidths are due to a combination of power broadening, short exposure duration, finite laser linewidth and laser frequency instability.

To achieve low uncertainty, it is important to understand the Zeeman shifts. The theoretical Zeeman shifts have been discussed in Extended Data tables 1–3 of ref. ^20^. We performed the spectroscopy at a finite, but small magnetic field so that it was possible to interrogate specific Zeeman components while maintaining their shifts small.

Further systematic shifts were also studied (Methods). Table 2 provides a summary of the determined shifts. Note that this is one of the first characterizations of the systematic shifts of a rovibrational transition of a homonuclear molecular ion. The deperturbed transition frequencies are ${f}_{a}^{({\rm{e}}{\rm{x}}{\rm{p}}{\rm{t}})}=124,487,067,172.9{(6)}_{{\rm{e}}{\rm{x}}{\rm{p}}{\rm{t}}}\,{\rm{k}}{\rm{H}}{\rm{z}}$ and ${f}_{b}^{({\rm{e}}{\rm{x}}{\rm{p}}{\rm{t}})}=124,486,980,347.5{(8)}_{{\rm{e}}{\rm{x}}{\rm{p}}{\rm{t}}}\,{\rm{k}}{\rm{H}}{\rm{z}}$.

**Table 2:** Error budget of the overtone (*v* = 1, *N* = 0) → (*v*′ = 3, *N*′ = 2) transition frequency of ${{\bf{H}}}_{{\bf{2}}}^{{\boldsymbol{+}}}$

	Component *a*_1_		Component *b*_1_		Component *b*_2_	
Effect	${{\boldsymbol{f}}}_{{{\boldsymbol{a}}}_{{\bf{1}}},{\bf{nom}}}^{({\boldsymbol{\mbox{--}}})}{\boldsymbol{\mbox{--}}}{{\boldsymbol{f}}}_{{\boldsymbol{a}}}^{({\bf{expt}})}$ (kHz)	Uncertainty (kHz)	${{\boldsymbol{f}}}_{{{\boldsymbol{b}}}_{{\bf{1}}},{\bf{nom}}}^{({\boldsymbol{\mbox{--}}})}{\boldsymbol{\mbox{--}}}{{\boldsymbol{f}}}_{{\boldsymbol{b}}}^{({\bf{expt}})}$ (kHz)	Uncertainty (kHz)	${{\boldsymbol{f}}}_{{{\boldsymbol{b}}}_{{\bf{2}}},{\bf{nom}}}^{({\boldsymbol{\mbox{--}}})}{\boldsymbol{\mbox{--}}}{{\boldsymbol{f}}}_{{\boldsymbol{b}}}^{({\bf{expt}})}$ (kHz)	Uncertainty (kHz)
Zeeman shift	80.2	0.46	120.02	0.68	39.87	0.23
a.c. Stark (2.4 μm)	−0.33	0.38	−0.35	0.41	−0.72	0.84
a.c. Stark (313 nm)	0.29	0.08	0.31	0.08	0.52	0.13
RF trap shift	0.05	0.06	0.05	0.07	0.11	0.14
AT splitting	−0.1	0.01	0	0.1	0	0.1
Recoil	17.07	0	17.07	0	17.07	0
Total	97.19	0.6	137.1	0.81	56.86	0.89

${f}_{a}^{({\rm{e}}{\rm{x}}{\rm{p}}{\rm{t}})}$ and ${f}_{b}^{({\rm{e}}{\rm{x}}{\rm{p}}{\rm{t}})}$ are the two deperturbed spin component frequencies given in the text and ${f}_{{a}_{1},{\rm{nom}}}^{(-)}$, ${f}_{{b}_{1},{\rm{nom}}}$ and ${f}_{{b}_{2},{\rm{nom}}}$ are the Zeeman components measured under nominal conditions shown in Figs. 1 and 2. ${f}_{{a}_{1},{\rm{nom}}}^{(-)}$ denotes the lower frequency component of the Autler–Townes (AT) doublet. See Methods, section ‘[Sec Sec14]’, for a detailed discussion of these effects.

## Comparison of experiment and theory

### Spin structure

From our analysis, we obtain the spin-rotation coefficient ${c}_{{\rm{e}}}^{({\rm{e}}{\rm{x}}{\rm{p}}{\rm{t}})}({v}^{{\prime} }=3,{N}^{{\prime} }=2)=34,730.18{(10)}_{{\rm{e}}{\rm{x}}{\rm{p}}{\rm{t}}}\,{\rm{k}}{\rm{H}}{\rm{z}}$. The most recent theoretical prediction is ${c}_{{\rm{e}}}^{({\rm{t}}{\rm{h}}{\rm{e}}{\rm{o}}{\rm{r}})}({v}^{{\prime} }=3,{N}^{{\prime} }=2)=34,730.25(12)\,{\rm{k}}{\rm{H}}{\rm{z}}$ (ref. ^5^). The values are consistent. We may compare this agreement with previous results. A more-than-50-years-old measurement of five spin-rotation coefficients in (*v* = 4, … 8; *N* = 2) levels^31^ is also in agreement with the theory but had substantially larger experimental uncertainties (about 1.5 kHz). Very precisely measured hyperfine splittings in levels (*v* = 4, 5, 6; *N* = 1) (ref. ^32^) agree with theoretical predictions within the 0.05 kHz theoretical uncertainties^5^ but do not allow extracting *c*_e_. Our measurement thus provides the most accurate determination, to our knowledge, of a spin structure coefficient of any MHI so far.

### Spin-averaged frequency

The adjusted, deperturbed experimental spin-averaged frequency is ${f}_{\text{spin-avg}}^{({\rm{e}}{\rm{x}}{\rm{p}}{\rm{t}})}=124,487,032,442.73{(0.95)}_{{\rm{e}}{\rm{x}}{\rm{p}}{\rm{t}}}\,{\rm{k}}{\rm{H}}{\rm{z}}$. Its fractional uncertainty 8 × 10^−12^ is 10^3^ times smaller than any previous experimental determination of a ${{\rm{H}}}_{2}^{+}$ property^20,23,32^. We included a correction for the recoil shift (17.1 kHz), as in our previous work^16^ (see Supplementary Information section E for details).

The theoretical transition frequency is computed as described in the Methods. To obtain a numerical value, a value for *m*_p_/*m*_e_ must be assumed. Committee on Data of the International Science Council (CODATA) 2018 provides such a value stemming only from Penning trap measurements (ESR and mass spectrometry), and it leads to ${f}_{\text{spin-avg}}^{({\rm{t}}{\rm{h}}{\rm{e}}{\rm{o}}{\rm{r}})}=124,487,032,$ $442.3{(1.0)}_{{\rm{QED}}}{(3.3)}_{{\rm{CODATA18}}}\,{\rm{kHz}}$.

Very recently, CODATA 2022 provided a more accurate new value [*m*_p_/*m*_e_]_22_, obtained by including some previous data of HD^+^ spectroscopy. Using this value yields $${f}_{\text{spin-avg}}^{({\rm{t}}{\rm{h}}{\rm{e}}{\rm{o}}{\rm{r}})}=124,487,032,442.5$$$${(1.0)}_{{\rm{Q}}{\rm{E}}{\rm{D}}}{(0.96)}_{{\rm{C}}{\rm{O}}{\rm{D}}{\rm{A}}{\rm{T}}{\rm{A}}22}\,{\rm{k}}{\rm{H}}{\rm{z}}$$. We point out that the two uncertainties in this number may be correlated. Both CODATA uncertainties are dominated by the uncertainty of the proton–electron mass ratio. Our experimental and theoretical values for *f*_spin-avg_ are in agreement.

## Discussion

### Frequency ratios

Ratios of transition frequencies of the same MHI species *X*, *f*_*i*_(*X*)/*f*_*j*_(*X*) have been introduced in ref. ^16^, and for different species *X* and *X*′ in ref. ^33^. They are illustrative quantities for comparison between experiment and theory, benefiting from the independence of the theoretical ratios from the Rydberg constant and its uncertainty. Ratios of suitably selected pairs *i* and *j* additionally have a reduced sensitivity to the mass ratios and charge radii. Taking into account the present rovibrational frequency of ${{\rm{H}}}_{2}^{+}$, ${f}_{{\rm{spin}}\text{-}{\rm{avg}}}({{\rm{H}}}_{2}^{+})\equiv {f}_{2}({{\rm{H}}}_{2}^{+})$ and our previously reported HD^+^ vibrational frequencies *f*_1_(HD^+^), *f*_5_(HD^+^) (ref. ^16^), we find that a favourable choice is ${{\mathcal{R}}}_{5,{2}^{{\prime} }}={f}_{5}({{\rm{HD}}}^{+})/{f}_{2}({{\rm{H}}}_{2}^{+})$, where transition *f*_5_ is the fourth vibrational overtone of HD^+^. We compare the experimental and theoretical ratio by considering their fractional deviation. The result is $({{\mathcal{R}}}_{5,{2}^{{\prime} }}^{{\rm{expt}}}-{{\mathcal{R}}}_{5,{2}^{{\prime} }}^{{\rm{theor}}})/{{\mathcal{R}}}_{5,{2}^{{\prime} }}^{{\rm{expt}}}=-\,2.7{(8.0)}_{{\rm{expt}}}{(0.5)}_{{\rm{theor}}}{(1.2)}_{{\rm{CODATA18* }}}\times 1{0}^{-12}$. The combined uncertainty is 8.1 × 10^−12^. For the frequency ratio ${f}_{1}({{\rm{H}}{\rm{D}}}^{+})/{f}_{2}({{\rm{H}}}_{2}^{+})$, we obtain a similar good match, $({{\mathcal{R}}}_{1,{2}^{{\prime} }}^{{\rm{e}}{\rm{x}}{\rm{p}}{\rm{t}}}-{{\mathcal{R}}}_{1,{2}^{{\prime} }}^{{\rm{t}}{\rm{h}}{\rm{e}}{\rm{o}}{\rm{r}}})/{{\mathcal{R}}}_{1,{2}^{{\prime} }}^{{\rm{e}}{\rm{x}}{\rm{p}}{\rm{t}}}\,=$ $1.0{(8.0)}_{{\rm{e}}{\rm{x}}{\rm{p}}{\rm{t}}}{(0.4)}_{{\rm{t}}{\rm{h}}{\rm{e}}{\rm{o}}{\rm{r}}}{(2.8)}_{{\rm{CODATA}}\mathrm{18* }}\times 1{0}^{-12}$. The asterisk symbol indicates that the recent accurate value ${[{m}_{{\rm{d}}}/{m}_{{\rm{p}}}]}_{{\rm{F}}21}$ (ref. ^6^) was used. At an operational level, assuming the correctness of the theoretical predictions, the match indicates that our measurements performed on different species, at different epochs and with in part differing equipment are consistent.

### Determination of fundamental constants

We may now determine some fundamental constants using least-squares adjustments (see Supplementary Information for details). We do not perform a global analysis of all world data, a task that is the mandate of CODATA, but limit ourselves to more restricted analyses that already provide important insights. An overview of our analyses of least-squares adjustments (LSA 1–LSA4) is presented in Table 3. We focus on the CODATA 2018 adjustment because it does not include results from MHI spectroscopy, thus allowing a clearer comparison with the present results.

**Table 3:** LSAs of fundamental constants

LSA with MHI data (CODATA 2018)											
Type	*n*	*M*	MHI input	Mass ratio input	Other input	Δ(*m*_e_/*m*_p_) *u*(*m*_e_/*m*_p_)	Δ(*m*_d_/*m*_p_) *u*(*m*_d_/*m*_p_)	Δ(*cR*_*∞*_), kHz *u*(*cR*_*∞*_), kHz	Δ(*r*_p_), fm *u*(*r*_p_), fm	Δ(*r*_d_), fm *u*(*r*_d_), fm	Comment
1	4	4	${f}_{{2}^{{\prime} }}$	–	*R*_*∞*,18_, *r*_p,18_	−1.6 × 10^−8^ 4.7 × 10^−8^					Adjusted *m*_p_/*m*_e_ to be compared with CODATA 2018 and with HD^+^ and CODATA 2018
2	9	8	${f}_{{2}^{{\prime} }},{f}_{1},{f}_{5}$	–	*R*_*∞*,18_, *r*_p,18_, *r*_d,18_	−2.2 × 10^−8^ 4.7 × 10^−8^	1.3 × 10^−10^ 1.1 × 10^−10^	−0.1 6.4	0 0.0019	0 0.00074	Adjusted *m*_d_/*m*_p_ to be compared with [*m*_d_/*m*_p_]_FM21_
3	12	10	${f}_{{2}^{{\prime} }},{f}_{1},{f}_{5}$	${[{m}_{{\rm{d}}}/{m}_{{\rm{p}}}]}_{{\rm{F}}{\rm{M}}21}$	H, H–D	−1.5 × 10^−7^ 1.0 × 10^−6^	1.2 × 10^−10^ 4.4 × 10^−9^	2.3 × 10^2^ 1.8 × 10^3^	0.08 0.58	0.03 0.23	Adjusted *m*_p_/*m*_e_, *R*_*∞*_ and *r*_p_ to be compared with CODATA 2018
4	10	8	${f}_{{2}^{{\prime} }},{f}_{1},{f}_{5}$	${[{m}_{{\rm{d}}}/{m}_{{\rm{p}}}]}_{{\rm{F}}{\rm{M}}21}$	*R*_*∞*,18_, *r*_p,18_, *r*_d,18_	−1.8 × 10^−8^ 3.4 × 10^−8^	1.2 × 10^−10^ 9.0 × 10^−12^	−0.1 6.4	0 0.0019	0 0.00074	Adjusted *m*_p_/*m*_e_ to be compared with CODATA 2018
**Reference values**											
CODATA 2022						−4.0 × 10^−9^ 3.2 × 10^−8^	−2.1 × 10^−12^ 8.4 × 10^−12^	−0.8 3.6	0 0.00064	0 0.00027	
CODATA 2018						– 1.1 × 10^−7^	– 9 × 10^−12^	– 6.4	– 0.0019	– 0.00074	
Reference ^6^, [*m*_d_/*m*_p_]_FM21_							– 9 × 10^−12^				
HD^+^	7	7	*f*_1_, *f*_5_	–	*R*_*∞*,18_, *r*_p,18_, *r*_d,18_	−4.6 × 10^−9^ 3.7 × 10^−8^		0 6.4	0 0.0019	0 0.00074	*m*_p_/*m*_e_ is computed from adjusted *μ*_pd_/*m*_e_ and [*m*_d_/*m*_p_]_FM21_

See text for a discussion and Supplementary Information for details. *n* is the number of input data; *M* is the number of adjusted constants; *f*_2′_ is short-hand for the frequency measured in the present work. Columns 7–11 refer to the adjusted fundamental constants. An exception is LSA HD^+^(bottom row), where *μ*_pd_/*m*_e_ is adjusted (but not shown) and from it and [*m*_d_/*m*_p_]_FM21_, the value ${[{m}_{{\rm{p}}}/{m}_{{\rm{e}}}]}_{{{\rm{HD}}}^{+}}$ is then computed. LSA HD^+^ concerns the molecule HD^+^ and is given for comparison. For the other LSAs, *m*_d_/*m*_p_ is derived from the adjusted *m*_p_/*m*_e_ and *μ*_pd_/*m*_e_. Also adjusted, but not shown, are the unknown theoretical corrections *δf*^(theor)^ to the currently available theoretical predictions *f*^(theor)^ of transition frequencies in MHI, of the 1*s*–2*s* transition in hydrogen (H) and of the 1*s*–2*s* hydrogen–deuterium isotope shift (H–D). *R*_*∞*,18_, *r*_p,18_, *r*_d,18_ denote the CODATA 2018 values. Δ(*x*) is the difference between the adjusted constant *x* and its CODATA 2018 value. *u*(*x*) is the uncertainty of the adjusted *x*, obtained from the LSA. The row ‘CODATA 2022’ shows Δ(*x*) values of the 2022 adjustment minus the 2018 adjustment values. However, we have replaced the CODATA 2018 value of *m*_d_/*m*_p_ with the more accurate [*m*_d_/*m*_p_]_FM21_. This is reflected in the Δ(*m*_d_/*m*_p_) and the *u*(*m*_d_/*m*_p_) values shown in the section “Reference values”.

The simplest analysis (LSA 1) consists of determining the proton–electron mass ratio. The CODATA 2018 Rydberg constant *R*_*∞*,18_ and the proton charge radius *r*_p,18_ are input data of the adjustment, although they are not effectively adjusted. We recall that both values are derived by combining the results of H and μH spectroscopy. The spectroscopically determined value is ${[{m}_{{\rm{p}}}/{m}_{{\rm{e}}}]}_{{\rm{L}}{\rm{S}}{\rm{A}}1}=1,836.152673414(47)$.

It is consistent with three other values:

(1) the CODATA 2018 value that relies mostly on an electron-*g*-factor measurement of hydrogen-like carbon, $${[{m}_{{\rm{p}}}/{m}_{{\rm{e}}}]}_{18}=1,836.15267343(11)$$ (ref. ^24^), whose uncertainty is 2.3 times the present one; (2) the CODATA 2022 value $${[{m}_{{\rm{p}}}/{m}_{{\rm{e}}}]}_{22}=1,836.152673426(32)$$ whose determination includes HD^+^ spectroscopy data and whose uncertainty is 1.4 times lower than the present one; and (3) the value $${[{m}_{{\rm{p}}}/{m}_{{\rm{e}}}]}_{{{\rm{H}}{\rm{D}}}^{+}}\,=\,$$$$1,836.152673425(37)$$ (LSA HD^+^ in Table 3), determined from our HD^+^ vibrational data, and *R*_*∞*,18_, *r*_p,18_, *r*_d,18_ and [*m*_d_/*m*_p_]_FM21_. It should be emphasized that the two CODATA determinations also rely on high-accuracy QED calculations.

The two values [*m*_d_/*m*_p_]_LSA1_ and ${[{m}_{{\rm{p}}}/{m}_{{\rm{e}}}]}_{{{\rm{HD}}}^{+}}$ from MHI spectroscopy are not independent in a fundamental sense, because they originate from the same apparatus, researchers, theoretical formalism and numerical routines (and are based on the same H/D, μH data and theory). The two corresponding uncertainties are partially correlated. Nevertheless, the agreement represents a powerful consistency test of our theoretical and experimental techniques.

LSA 2 combines the present ${{\rm{H}}}_{2}^{+}$ data with our previous HD^+^ rovibrational data, CODATA values *R*_*∞*,18_, *r*_p,18_ and *r*_d,18_ but does not include mass data. Spectroscopic values both for *m*_p_/*m*_e_ and *m*_d_/*m*_p_ are obtained. The value of the latter is [*m*_d_/*m*_p_]_LSA2_ = 1.99900750140(11). It is in agreement with the independent value [*m*_d_/*m*_p_]_FM21_ = 1.999007501272(9) from mass spectrometry^6^.

With LSA 3, we check whether *R*_*∞*_, *r*_p_, *r*_d_ and *m*_p_/*m*_e_ can be obtained with a useful level of uncertainty without relying on input data derived from muonic hydrogen experiments. Therefore, instead of CODATA 2018 fundamental constants, we use specific data from H and D spectroscopy, the 1*s*–2*s* transition frequencies^34–36^, as supplementary input, apart from the accurate [*m*_d_/*m*_p_]_FM21_. Note that the H–D isotope shift data furnishes, by itself, a highly accurate value of ${r}_{{\rm{d}}}^{2}-{r}_{{\rm{p}}}^{2}$ (ref. ^34^). As a result of LSA 3, the proton radius is determined to be [*r*_p_]_LSA3_ = 0.92(58) fm. Its uncertainty is not competitive. The weakness of LSA 3 can be traced to the current experimental uncertainties and the similar fractional sensitivities of the MHI frequencies on the fundamental constants (see ref. ^33^ for details).

In LSA 4, an extension of LSA 2, we combine our MHI data with [*m*_d_/*m*_p_]_FM21_, *R*_*∞*__,18_ *r*_p,18_ and *r*_d,18_. The adjusted proton–electron mass ratio is slightly more accurate than in LSA 1, with an uncertainty three times lower than in CODATA 2018. The value is consistent with the CODATA 2022 value (which includes *f*_1_(HD^+^) in the adjustment).

## Conclusion

This work is one of the first demonstrations of high-accuracy rovibrational spectroscopy of a homonuclear molecular ion, and it introduces ${{\rm{H}}}_{2}^{+}$ into the field of fundamental constants, resulting in the spectroscopic determination of the proton–electron mass ratio at a state-of-the-art level. The value is in agreement but exhibits more than two-fold lower uncertainty than the value obtained from the *g*-factor of hydrogen-like carbon. Our value is also in agreement with the *m*_p_/*m*_e_ value obtained by combining HD^+^ laser spectroscopy and a mass spectrometric determination of *m*_d_/*m*_p_.

Furthermore, we performed a stringent test of a specific aspect of MHI spin structure theory, namely, the spin–rotation coupling. The obtained agreement between experiment and theory supports the suitability of current spin structure theory to the analysis of experimental HD^+^ data.

Furthermore, we deduced a new, spectroscopically determined value for the deuteron–proton mass ratio. This value relies on spectroscopy and on the QED theory of the hydrogen atom, the muonic hydrogen atom and the MHI. The most accurate value for comparison is from an experiment in which an ${{\rm{H}}}_{2}^{+}$ ion and a deuteron were in classical motion in a Penning trap, with only minor quantum corrections applied in the ratio extraction^6^. The two independent *m*_d_/*m*_p_ values agree at a fractional level of 5.4 × 10^−11^. Therefore, this represents a strong test of the correct description of dynamics in the quantum and classical realms: the mass values in the Schrödinger equation are the same as those of Newtonian physics.

Finally, the finding that the two ratios of experimental and theoretical frequencies of HD^+^ and ${{\rm{H}}}_{2}^{+}$ agree at the 8.1 × 10^−12^ level (limited by the present experimental uncertainty) ranks among the most accurate comparisons of a theoretical prediction and an experimental quantity. The uncertainty is within a factor of 8 of the most accurate comparison of experiment and theory, the *g*-factor of the bare electron^37^. Near-future experimental improvements may enable the reduction of uncertainties associated with particular ratios to a comparable level^4^.

## Outlook

In MHI research, it is desirable to pursue even higher accuracy on ${{\rm{H}}}_{2}^{+}$ and extension to the remaining homonuclear ions, ${{\rm{D}}}_{2}^{+}$ and ${{\rm{T}}}_{2}^{+}$. According to a new analysis^4^, important improvements in the accuracy of mass ratios (more than 100-fold compared with CODATA 2022) and of proton, deuteron and triton nuclear charge radii are feasible. Our results (Methods) indicate that a more than ten-fold lower experimental uncertainty is possible with our methods, by removing the uncertainty associated with the limited number of measured Autler–Townes doublets and by an improved determination of a.c. Stark shifts. We showed that by selecting a suitable transition and measuring all its spin components, it is possible to remove the effect of spin structure entirely in the determination of the spin-averaged frequency, thus avoiding the use of spin theory results. This is an important advantage compared with HD^+^. MHI spectroscopy at even lower uncertainty levels is possible with quantum logic spectroscopy^38^, as proposed early on^39^. This technique has already been applied to molecular ions^40–43^ and most recently to ${{\rm{H}}}_{2}^{+}$ (ref. ^44^). Beyond MHI, our demonstration also supports efforts of using homonuclear diatomic ions for further fundamental physics studies, in particular, for testing the time-independence of the electron–nuclear mass ratio^39,45–47^.

The anticipated future improvement of the proton–electron mass ratio may have a substantial impact on ESR spectrometry in Penning traps. There, one measures the ESR frequency *ν*_L_(*B*) and the cyclotron frequency *ν*_c_(*B*) of one-electron ions in the same magnetic field *B*. These ions can be hydrogen-like ions (HLI) or MHI, with mass *m*_ion_, charge *q*_ion_ and bound-electron *g*-factor *g*_ion_. Combining the two frequencies provides *m*_e_/*m*_ion_ = (*g*_ion_/2)(*e*/*q*_ion_)(*ν*_c_(*B*)/*ν*_L_(*B*)). The proton–electron mass ratio [*m*_p_/*m*_e_]_MHI_ from MHI vibrational spectroscopy may be used to develop the expression into ${g}_{{\rm{i}}{\rm{o}}{\rm{n}}}=2{[{m}_{{\rm{e}}}/{m}_{{\rm{p}}}]}_{{\rm{M}}{\rm{H}}{\rm{I}}}({m}_{{\rm{p}}}/{m}_{{\rm{i}}{\rm{o}}{\rm{n}}})({q}_{{\rm{i}}{\rm{o}}{\rm{n}}}/e)({\nu }_{{\rm{L}}}/{\nu }_{{\rm{c}}})$. Since the baryon mass ratio *m*_p_/*m*_ion_ can be measured separately and with high accuracy by Penning trap cyclotron mass spectrometry, this expression then allows us to compare the experimental values ${g}_{{\rm{ion}}}^{({\rm{expt}})}$ with the predicted values ${g}_{{\rm{ion}}}^{({\rm{theor}})}$, for example, for testing strong-field QED in highly-charged HLI. As near-future MHI vibrational spectroscopy and theory may achieve ${u}_{{\rm{r}}}({[{m}_{{\rm{e}}}/{m}_{{\rm{p}}}]}_{{\rm{MHI}}})\simeq 1\times 1{0}^{-13}$ (ref. ^4^), *g*-factor determinations and *g*-factor-based QED tests may, in principle, become possible at the same level. Alternatively, using theoretical *g*-factors, mass ratios *m*_p_/*m*_ion_ may be determined without the necessity of conducting a measurement on the proton.

A future test of CPT symmetry consists of the comparison of a single vibrational transition frequency of anti-${{\rm{H}}}_{2}^{+}$ with the same in normal ${{\rm{H}}}_{2}^{+}$. In refs. ^1,3,20,48–50^, the motivation and accuracy potential for such a test have been discussed. The present work represents progress towards this goal, because E2 spectroscopy is equally applicable to anti-${{\rm{H}}}_{2}^{+}$. Although the present work was performed in an RF trap, future anti-${{\rm{H}}}_{2}^{+}$ spectroscopy might be performed in a Penning trap. The recent demonstration of long-term trapping and non-destructive spectroscopy of HD^+^ in a Penning trap supports this approach^51^. New Penning trap techniques are under development^52^.

## Methods

### Details of the experiment

We use the same apparatus that we used in our previous work^20^ to perform laser spectroscopy of an E2 transition in ${{\rm{H}}}_{2}^{+}$ and HD^+^. We prepare a cluster of trapped and sympathetically cooled molecular ions, in which the sympathetic cooling is provided by the Coulomb interaction between the molecular ions and the three-dimensional cluster of laser-cooled Be^+^ ions. By loading a small number of molecular ions, they arrange as an ion chain extending along the symmetry axis of the ion trap. The loading occurs by electron impact ionization from background para-H_2_ gas. When the direction of propagation of the spectroscopy beam is perpendicular to the chain, a Doppler-free profile can be observed^16^.

A partial state preparation of the trapped ${{\rm{H}}}_{2}^{+}$ ions is carried out by dissociating those in excited vibrational levels *v* ≥ 2 (ref. ^20^), using two lasers at 313 nm and 405 nm. We emphasize that our method does not prepare the population in a specific rotational level.

Following the state preparation, the number of trapped ${{\rm{H}}}_{2}^{+}$ ions is determined by recording the beryllium fluorescence signal accompanying the transverse secular excitation of ${{\rm{H}}}_{2}^{+}$, as a function of excitation frequency. The peak strength of this secular spectrum is proportional to the number of trapped ions. Next, the MHI are subjected to resonance-enhanced, multi-photon dissociation (REMPD). It comprises the excitation of the spectroscopy transition by an optical parametric oscillator (OPO) wave and the dissociation from the upper spectroscopy level by a 405-nm laser wave. To prevent a light shift induced by the dissociation laser, an interleaved shuttering scheme is used. The cycle is concluded with an assessment of the remaining trapped ${{\rm{H}}}_{2}^{+}$ ions. Subsequent cycles alternate between spectroscopy and background measurements (in which the spectroscopy laser is blocked) until sufficient statistics have been gathered. Between cycles, the ion cluster is purged of ions other than Be^+^ and a new ${{\rm{H}}}_{2}^{+}$ ensemble is loaded to ensure that the lower spectroscopy level has sufficient population. Throughout the REMPD, a magnetic field of *B*_REMPD_ ≃ 7.14(4) μT was applied, determined as explained in a later section. The spectroscopic signal is derived by comparing the number of trapped ${{\rm{H}}}_{2}^{+}$ ions before and after REMPD. This is computed as the normalized decrease in the ion number.

The spectroscopy laser system has been described previously^20^. We use the idler wave of the OPO as the spectroscopy wave that is stabilized in frequency by referencing it to an ultrastable optical frequency comb^27^. The upper-bound linewidth of the idler wave depends on the reference laser used for the optical frequency comb. Over the course of the data acquisition, two different reference lasers were used. Characterization showed that they produce a spectroscopy wave linewidth of approximately 5 Hz and 20 Hz, respectively, on timescales of 1–10^3^ s. The measurements acquired under nominal conditions and at high-trap RF amplitude were taken with a 20 Hz linewidth. All other systematic measurements (Supplementary Information) were obtained with a 5-Hz spectroscopy wave linewidth. As this linewidth is moderately smaller than the observed linewidths, it seems to contribute to the observed molecular transition linewidths. For long-term frequency stability, the optical frequency comb is referenced to a hydrogen maser. Furthermore, we compare our hydrogen maser with the German national standard using Global Navigation Satellite System (GNSS), thereby ensuring Système-International-traceability of the frequency of the spectroscopy wave. The combined frequency error of the spectroscopy wave for timescales of one REMPD cycle is ≤1 Hz, or ≤1 × 10^−14^ in relative terms. This includes fluctuations of the laser, statistical errors of the frequency comb measurement and maser frequency corrections (see section ‘Maser shift’).

We deliberately performed a blind experimental search of the transitions, using a value of ${f}_{\text{spin-avg}}^{({\rm{theor}})}$ as input information with an added offset that turned out to be approximately 80 kHz after unblinding. The narrow linewidth of the transitions and the large Zeeman shifts of the components rendered their discovery tedious. ${f}_{\text{spin-avg}}^{({\rm{theor}})}$ as reported above was disclosed only after completion of all measurements. Its value was taken into account to identify the observed transitions, which is a necessary step for performing the complete analysis.

### Systematics of the ${{\bf{H}}}_{{\bf{2}}}^{{\boldsymbol{+}}}$ E2 transition

We have measured all systematics effects that we believe to be of significant magnitude compared with our spectroscopic resolution. For each shift, the measurements have been done on a subset of the three Zeeman components ${f}_{{a}_{1}}$, ${f}_{{b}_{1}}$ and ${f}_{{b}_{2}}$. Figures of the corresponding lines are shown in the Supplementary Information. In total, we have measured 11 individual lines, 4 of which were under nominal conditions (Fig. 2). The remaining lines were Zeeman components perturbed by a single parameter setting (a laser intensity or the RF amplitude of the trap). To evaluate all systematic effects as well as ${f}_{\text{spin-avg}}^{({\rm{e}}{\rm{x}}{\rm{p}}{\rm{t}})}$ and ${c}_{{\rm{e}}}^{({\rm{expt}})}$ we performed an adjustment to all observed lines (see section ‘Evaluation of deperturbed values ${f}_{\text{spin-avg}}^{({\rm{expt}})}$ and ${c}_{{\rm{e}}}^{({\rm{expt}})}$’). Below, we first enumerate the shifts that we considered and subsequently discuss the adjustment.

#### Zeeman shift

To determine the Zeeman shifts of the components, we rely on theoretical results^28,53^. The three measured components have substantial linear and small quadratic shifts. The predicted shift coefficients are ${c}_{{\rm{l}}{\rm{i}}{\rm{n}},{a}_{1}}=11.2\,{\rm{k}}{\rm{H}}{\rm{z}}\,{{\rm{\mu T}}}^{-1}$, ${c}_{{\rm{l}}{\rm{i}}{\rm{n}},{b}_{1}}=16.8\,{\rm{k}}{\rm{H}}{\rm{z}}\,{{\rm{\mu T}}}^{-1}$, ${c}_{{\rm{l}}{\rm{i}}{\rm{n}},{b}_{2}}=5.6\,{\rm{k}}{\rm{H}}{\rm{z}}\,{{\rm{\mu T}}}^{-1}$, ${c}_{{\rm{q}}{\rm{u}}{\rm{a}}{\rm{d}},{a}_{1}}=2.2\,{\rm{k}}{\rm{H}}{\rm{z}}\,{{\rm{\mu T}}}^{-2}$, ${c}_{{\rm{q}}{\rm{u}}{\rm{a}}{\rm{d}},{b}_{1}}=-2.2\,{\rm{k}}{\rm{H}}{\rm{z}}\,{{\rm{\mu T}}}^{-2}$, ${c}_{{\rm{q}}{\rm{u}}{\rm{a}}{\rm{d}},{b}_{2}}=-1.4\,{\rm{k}}{\rm{H}}{\rm{z}}\,{{\rm{\mu T}}}^{-2}$. In section ‘[Sec Sec22]’, we use these coefficients as input parameters in the determination of the Zeeman shifts and treat the magnetic field value *B* as an adjustable parameter. The adjusted value *B*_REMPD_ ≃ 7.14(4) μT is in agreement with, but more precise than, the value determined in our previous spectroscopy experiments on HD^+^.

As our total measurement duration extended over several months, the long-term stability of the magnetic field was verified. Measurements of one line, performed a few months apart, yielded no observable frequency shift within experimental uncertainty. Considering the above Zeeman coefficients, we deduce a mean drift rate below 19 pT day^−1^. This bound has an insignificant impact compared with our overall measurement uncertainty.

#### The a.c. and d.c. Stark shifts

During the REMPD, two laser waves were present: the spectroscopy wave (2.4 μm) and the Doppler cooling wave (313 nm). These cause a.c. Stark shifts (light shifts) of the transition frequencies.

We have, therefore, measured the component ${f}_{{a}_{1}}$ at two different intensities of the spectroscopy wave (Supplementary Fig. 2). The a.c. Stark shift of other components are determined relative to this measurement by making use of the ratios of theoretically calculated polarizabilities (see section ‘[Sec Sec22]’).

We remark that alternatively, an estimate for the shift can be obtained from the theoretical polarizabilities^54^ and an approximate value for the spectroscopy wave intensity. This estimate gives maximum 2.4-μm-light shifts of the order of −60 Hz for both components *f*_*a*_ and *f*_*b*_, consistent with our observation, but one order smaller than the bounds resulting from our evaluation (Table 2). However, we take a conservative approach, relying more on our experimental data.

Regarding the shift caused by the 313-nm wave, we have measured two components, ${f}_{{a}_{1}}$ and ${f}_{{b}_{1}}$, at three intensities each. The more detailed investigation of this shift was motivated by the large observed dependence on 313 nm wave intensity. For this wave, we similarly use theoretical polarizabilities to infer the shifts not directly measured.

Moreover, we assume a linear dependence of the transition frequencies on both wave intensities, with common sensitivity parameters *k*_313_ and *k*_2.4_ for all components (see section ‘[Sec Sec22]’).

We have not measured the shift of the transition frequency arising from a spatial offset of the ${{\rm{H}}}_{2}^{+}$ ensemble relative to the nominal location in the trap. This effect is expected to be negligible, given that the trap is well compensated and that the residual static electric field is small. This expectation is supported by the fact that we have previously investigated the d.c. Stark effect in HD^+^ E2 transitions, and we have not resolved any shift at the level of 90 Hz (ref. ^20^), and that the static polarizability of the present ${{\rm{H}}}_{2}^{+}$ transition is more than two orders of magnitude smaller than that of the HD^+^ transition^54^. For these reasons, we expect the trap offset effect to be negligible and assign it a zero value and uncertainty.

#### Radiofrequency trap shift

We have measured the transition frequency ${f}_{{a}_{1}}$ at two different trap RF amplitudes. In contrast to HD^+^, in which for an E2 transition we observed shifts of approximately 1.2 kHz for two equivalent Zeeman components of the studied spin component^20^, here we did not resolve a shift. Although a possible shift would increase with increasing RF amplitude, we were unable to perform measurements at RF amplitudes larger or smaller than those presented, because the background loss of the trapped ${{\rm{H}}}_{2}^{+}$ ensemble then increases. The non-observation of a shift confirms our earlier hypothesis^20^ that the trap-field-induced shift is smaller for homonuclear MHIs compared with heteronuclear MHIs, because of the absence of off-resonant electric dipole coupling between each spectroscopy level and other rovibrational levels. However, electric dipole couplings to excited electronic states of ${{\rm{H}}}_{2}^{+}$ are nonzero. Therefore, a shift could still occur, but of much smaller magnitude. Therefore, we model the RF trap shift, similarly to the a.c. Stark shifts above, by the use of theoretical polarizabilities, an overall sensitivity parameter *k*_RF_ and a quadratic dependence on the RF amplitude of the trap. The quadratic dependence is known from previous experiments^20^.

#### Autler–Townes splitting or a.c. Stark splitting

The upper spectroscopy level interacts with two light fields, 2.4 μm and 313 nm, where the latter couples to the continuum. We, therefore, observe a splitting of the line ${f}_{{a}_{1}}$. This is known as the Autler–Townes effect (or a.c. Stark splitting)^55^ and has earlier been observed for multi-photon processes in strong laser fields, both continuous-wave and pulsed. For the case of HD^+^, we have previously investigated this effect in our apparatus and determined a square-root dependence on the UV-laser intensity (see Supplementary Information section E), consistent with reports in the literature^56,57^. We have measured the splitting of component ${f}_{{a}_{1}}$, for nominal and high trap RF amplitudes. Both observed splittings agree within the combined uncertainties. The mean amounts to $\Delta {f}_{{\rm{AT,nom}},{a}_{1}}=195(15)\,{\rm{Hz}}$ at the nominal 313 nm intensity *I*_313,nom_. We assume that the ratio $\Delta {f}_{{\rm{AT}}}/\sqrt{{I}_{313}}=\Delta {f}_{{\rm{AT}},{\rm{nom}},{a}_{1}}/\sqrt{{I}_{313,{\rm{nom}}}}$ is the same for all lines.

#### Black-body radiation shift

The black-body radiation shift of ${{\rm{H}}}_{2}^{+}$ is predicted to be of the order of 10^−17^ fractionally at room temperature^54^ and is ignored.

#### Maser shift

As in our previous works^13,15,16,20^, the frequency of the spectroscopy wave is measured relative to the 5 MHz output of a hydrogen maser. This frequency is continuously compared with a 1 pulse-per-second signal provided by a GNSS receiver. Common-view GNSS data allow us to determine the maser frequency with respect to the German national time standard. We determined the fractional frequency offset of the maser to be approximately +1 × 10^−11^, and the fractional drift was approximately +3 × 10^−15^ day^−1^. The measured laser frequencies are corrected for the time-varying maser offset. The uncertainty of this correction was determined to be approximately 10 mHz.

#### Recoil shift

This is discussed in Supplementary Information section E.3.

#### Evaluation of deperturbed values ${{\boldsymbol{f}}}_{{\bf{s}}{\bf{p}}{\bf{i}}{\bf{n}}{\boldsymbol{\mbox{--}}}{\bf{a}}{\bf{v}}{\bf{g}}}^{{\bf{(e}}{\bf{x}}{\bf{p}}{\bf{t)}}}$ and ${{\boldsymbol{c}}}_{{\bf{e}}}^{{\bf{(e}}{\bf{x}}{\bf{p}}{\bf{t)}}}$

The determination of the quantities of interest in the presence of the above systematic effects is performed by an LSA. To this end, we model a measured transition frequency *j* as follows:

$${f}_{{\rm{o}}{\rm{b}}{\rm{s}},i}^{(j)}\doteq \,{f}_{\text{spin-avg}}^{({\rm{e}}{\rm{x}}{\rm{p}}{\rm{t}})}+{c}_{{\rm{s}}{\rm{p}}{\rm{i}}{\rm{n}},i}{c}_{{\rm{e}}}^{({\rm{e}}{\rm{x}}{\rm{p}}{\rm{t}})}+{c}_{{\rm{l}}{\rm{i}}{\rm{n}},i}{B}_{{\rm{R}}{\rm{E}}{\rm{M}}{\rm{P}}{\rm{D}}}+{c}_{{\rm{q}}{\rm{u}}{\rm{a}}{\rm{d}},i}{B}_{{\rm{R}}{\rm{E}}{\rm{M}}{\rm{P}}{\rm{D}}}^{2}+\delta {f}_{{\rm{A}}{\rm{T}}}^{(j)}+{\Sigma }_{{\rm{e}}{\rm{f}}{\rm{f}}{\rm{e}}{\rm{c}}{\rm{t}}}\delta {f}_{{\rm{e}}{\rm{f}}{\rm{f}}{\rm{e}}{\rm{c}}{\rm{t}},i}^{(j)}\,.$$

The dotted equality sign means that the left and right hand sides should agree within estimated uncertainties. The subscript *i* refers to both a particular spin-rotation component and a particular Zeeman component. The index *j* denotes individual measurements, that is, for a given *i*, the superscript *j* may take on different values. ${f}_{{\rm{obs}},i}^{(j)}$ is the measured line frequency with a statistical uncertainty given by the half width at half maximum of the line and $\delta {f}_{{\rm{A}}{\rm{T}}}^{(j)}$ is its shift due to the Autler–Townes effect. The spin coefficients of the upper spectroscopy level are ${c}_{{\rm{spin}},i}=({F}_{i}^{{\prime} }({F}_{i}^{{\prime} }+1)-N(N+1)-3/4)/2$ (see main text) and the Zeeman coefficients *c*_lin,*i*_, *c*_quad,*i*_ of component *i* are taken to be the theoretical values (see section ‘Zeeman shift’).

For each systematic shift, we define the contribution as $$\delta {f}_{{\rm{effect}},i}^{(j)}\,=\,$$$${r}_{{\rm{effect}},i}{k}_{{\rm{effect}}}{Q}_{{\rm{effect}}}^{(j)}$$, with the relative sensitivity *r*_effect,*i*_ of a spin-rotation Zeeman component *i* and the global sensitivity *k*_effect_ of the rovibrational transition. The parameter $${Q}_{{\rm{e}}\mathrm{ff}ect}^{(j)}$$ is one of the following: the spectroscopy wave power, $${P}_{2.4}^{(j)}$$, the Doppler cooling wave power $${P}_{313}^{(j)}$$ or the squared trap RF amplitude $${({V}_{{\rm{R}}F}^{(j)})}^{2}$$. The parameters *k*_effect_ must be adjusted by the LSA, because we do not know the precise light intensities and electric fields at the locations of the ions.

The values *r*_effect,*i*_ are computed as the ratios of theoretical polarizabilities of the Zeeman components^54^. The total polarizability of a component is the sum of a scalar (spin-independent) polarizability *α*_s_ and a tensor polarizability, where the latter can be expressed as a spin-independent value *α*_t_ multiplied by a state-dependent factor *S* and a polarization-dependent factor *G*. The tensor polarizability is zero when *N* = 0; hence, it is zero in the lower spectroscopic states. We have $S({f}_{{a}_{1}}\,)=-\,24/5$, $S({f}_{{b}_{1}}\,)=-\,21/5$ and $S({f}_{{b}_{2}}\,)=21/5$. To compute the total polarizability of the transitions, it is necessary to combine the values of the scalar and tensor polarizabilities of the lower and upper levels. Note that the *r*_*i*,effect_ of the same component *i* are distinct for different effects, because the scalar and tensor polarizabilities are, in general, frequency-dependent. For the case of the RF and 2.4 μm fields, the polarizabilities can be found in refs. ^20,54^, respectively, whereas for the 313 nm field, we have computed them to be *α*_s_(*v*′ = 3, *N*′ = 2) − *α*_s_(*v* = 1, *N* = 0) = 6.9 a.u. and *α*_t_(*v*′ = 3, *N*′ = 2)= −1.3 a.u. A table of numerical values *r*_effect,*i*_ used in the adjustment can be found in Supplementary Table 1.

To perform the LSA, the equations should be linearized. The only non-linear contribution is the quadratic Zeeman shift. We linearize the equations by expressing the magnetic field *B*_REMPD_ = *B*_0_ + *δB* as the sum of a constant value *B*_0_, approximately known from previous experiments, and an adjustable small deviation *δB*. The term quadratic in *δB* may safely be neglected.

In summary, the LSA includes 11 observational equations to which six parameters are adjusted, ${f}_{\text{spin-avg}}^{({\rm{expt}})}$, ${c}_{{\rm{e}}}^{({\rm{expt}})}$, *δB*, *k*_2.4_, *k*_313_ and *k*_RF_. All input data are uncorrelated.

The Autler–Townes effect is not considered in the same form as the other systematic effects, because it is a splitting rather than a shift. For those lines, for which we have measured the splitting (note that for these ${I}_{313}^{(j)}={I}_{313,{\rm{nom}}}$), the shift is simply given by half the observed splitting, $\delta {f}_{{\rm{AT}}}^{(j)}=\mp 1/2\Delta {f}_{{\rm{AT}},{\rm{nom}}}$. Positive and negative signs correspond to the smaller and larger frequency components, respectively. For the other lines, we present two approaches for accounting for the effect.

In approach 1, as the sign of $\delta {f}_{{\rm{A}}T}^{(j)}$ is unknown for these cases, we set $\delta {f}_{{\rm{A}}T}^{(j)}=0$, but with uncertainty $u(\delta {f}_{{\rm{A}}T}^{(j)})=\Delta {f}_{{\rm{A}}T}^{(j)}/2$. Furthermore, the scaling with intensity is $\Delta {f}_{{\rm{AT}}}^{(j)}=\sqrt{{I}_{313}^{(j)}/{I}_{313,{\rm{nom}}}}\times \Delta {f}_{{\rm{AT}},{\rm{nom}},{a}_{1}}$. As $\Delta {f}_{{\rm{AT}},{\rm{nom}},{a}_{1}}$ is much larger than the statistical uncertainty of the lines, and combined with the fact that our measurements can resolve a shift neither due to *I*_2.4_ nor due to *V*_RF_, the resulting uncertainty of ${{f}}_{{\rm{s}}{\rm{p}}{\rm{i}}{\rm{n}}\,-\,{\rm{a}}{\rm{v}}{\rm{g}}}^{({\rm{e}}{\rm{x}}{\rm{p}}{\rm{t}})}$ and ${c}_{{\rm{e}}}^{({\rm{e}}{\rm{x}}{\rm{p}}{\rm{t}})}$ far exceeds our line resolution. The values presented in the main text result from this approach. The values of systematic shifts of individual Zeeman components $\delta {f}_{{\rm{e}}{\rm{ff}}{\rm{e}}{\rm{c}}{\rm{t}}}^{(j)}$ given in Table 2 are computed using the resulting sensitivities *k*_effect_.

Approach 2 is described in the Supplementary Information.

### Ab initio theory of *f*_spin-avg_

Theoretical data have been obtained in two steps. First, the spin-averaged transition frequency was calculated as an expansion in terms of the coupling parameter, the fine structure constant *α*. We started from the nonrelativistic solution of the Schrödinger equation. Second, higher-order corrections were obtained in a perturbative way along the lines of the NRQED effective field theory^7^. The individual contributions are: *f*^(0)^ = 124,485,554,550.71 kHz (nonrelativistic three-body Schrödinger solution), *f*^(2)^ = 2,002,698.73 kHz (relativistic corrections in the Breit–Pauli approximation; nuclear radii), *f*^(3)^ = −521,345.53 kHz (leading-order one-loop radiative corrections), *f*^(4)^ = −3,689.05 kHz (one- and two-loop radiative corrections; relativistic corrections), *f*^(5)^ = 228.67 kHz (radiative corrections up to three loops, Wichmann–Kroll contribution), *f*^(6)^ = −1.62 kHz (one- and two-loop radiative diagrams, Wichmann–Kroll contribution), *f*^other^ = 0.54 kHz (muon and hadron vacuum polarization). Here, *f*^(*n*)^ denotes a contribution proportional to *cR*_*∞*_*α*^*n*^. The sum of all these contributions, ${f}_{\text{spin-avg}}^{({\rm{theor}})}$, together with the theoretical uncertainty, is given in the main text. The above values are for CODATA 2022.

The sensitivity of the spin-averaged frequency to *m*_p_/*m*_e_ has been reported in ref. ^20^, ∂*f*_spin-avg_/∂(*m*_p_/*m*_e_) = −0.43976 × *f*_spin-avg_/(*m*_p_/*m*_e_).

In the computation of the QED theory uncertainty of the frequency ratios, correlation coefficients and uncertainties proposed by J.-Ph. Karr have been used.

### Ab initio theory of the spin–rotation coupling

${c}_{{\rm{e}}}^{({\rm{theor}})}$, given in the main text, is calculated theoretically using the Breit–Pauli Hamiltonian and then including higher-order corrections up to order *mα*^7^ln *α*. When higher-order corrections are considered, other spin interaction terms may also appear, for example, proportional to **I**_1_ ⋅ **I**_2_. First, they are a factor ${\alpha }^{2}{({m}_{{\rm{e}}}/{m}_{{\rm{p}}})}^{2}$ smaller than the leading-order hyperfine structure splitting of the state. Second, the total nuclear spin in every rotational state *N* is fixed and thus also the value of **I**_1_ ⋅ **I**_2_ is uniquely determined. This means that these new terms do not contribute to the splitting, but to the spin-averaged energy. They are included in *f*^(4)^ above.

### Ab initio theory of the Zeeman interaction

The interaction of para-${{\rm{H}}}_{2}^{+}$ with an external magnetic field is described by the effective Hamiltonian:

$${H}_{{\rm{mag}}}=-\,{\mu }_{{\rm{B}}}({g}_{N}{\bf{N}}+{g}_{{\rm{e}}}{{\bf{S}}}_{{\rm{e}}})\cdot {\bf{B}},$$

where *μ*_B_ = |*e*|*ħ*/(2*m*_e_*c*) is the Bohr magneton, *g*_*N*_(*v*, *N*) is the orbital *g*-factor and *g*_e_(*v*, *N*) ≃ −2.002319 is the bound-electron *g*-factor. The anisotropy of *g*_e_ is neglected. *g*_*N*_ is calculated numerically from the nonrelativistic three-body-bound-state wave function^58^. For the upper level of the transition, *g*_*N*_(*v* = 3, *N* = 2) = 0.48156 × 10^−3^. The computation of the Zeeman shifts is given in the Supplementary Material of ref. ^1^.

## Online content

Any methods, additional references, Nature Portfolio reporting summaries, source data, extended data, supplementary information, acknowledgements, peer review information; details of author contributions and competing interests; and statements of data and code availability are available at 10.1038/s41586-025-09306-2.

## Supplementary information

Supplementary Information This file contains Supplementary Figs. 1–7, Supplementary Table 1 and Supplementary References.

Supplementary Data This file contains Source data for Supplementary Figs. 1–4 and 6.

## Source data

Source Data Fig. 2

## Data Availability

Source data are provided with this paper. All other data that support the plots within this paper and other findings of this study are available from the corresponding author upon reasonable request.

## Code availability

No custom code or software was used for the analysis and presentation of the data associated with this paper.
