# Supplementary material for: High-accuracy laser spectroscopy of H2+ and the proton–electron mass ratio
Source: Nature. 2025 Aug 6;644(8075):69–75. doi: 10.1038/s41586-025-09306-2 (PMC12328222; doi:10.1038/s41586-025-09306-2)
Supplement: Supplementary file 1 — This file contains Supplementary Figs. 1–7, Supplementary Table 1 and Supplementary References. [file 41586_2025_9306_MOESM1_ESM.pdf]

nature portfolio

https://doi.org/10.1038/s41586-025-09306-2

Supplementary information

High-accuracy laser spectroscopy of H_2^+ and the proton–electron mass ratio

In the format provided by the
authors and unedited

High-accuracy laser spectroscopy of H_2^+ and the proton-electron mass ratio

Supplementary Information

S. Alighanbari, M. R. Schenkel, and S. Schiller*

Heinrich-Heine-Universität Düsseldorf,

Mathematisch-Naturwissenschaftliche Fakultät,

Institut für Experimentalphysik, 40225 Düsseldorf, Germany

V. I. Korobov

Bogoliubov Laboratory of Theoretical Physics,

Joint Institute for Nuclear Research, 141980 Dubna, Russia

SUPPLEMENTARY INFORMATION

A. Figures of measurements of systematics

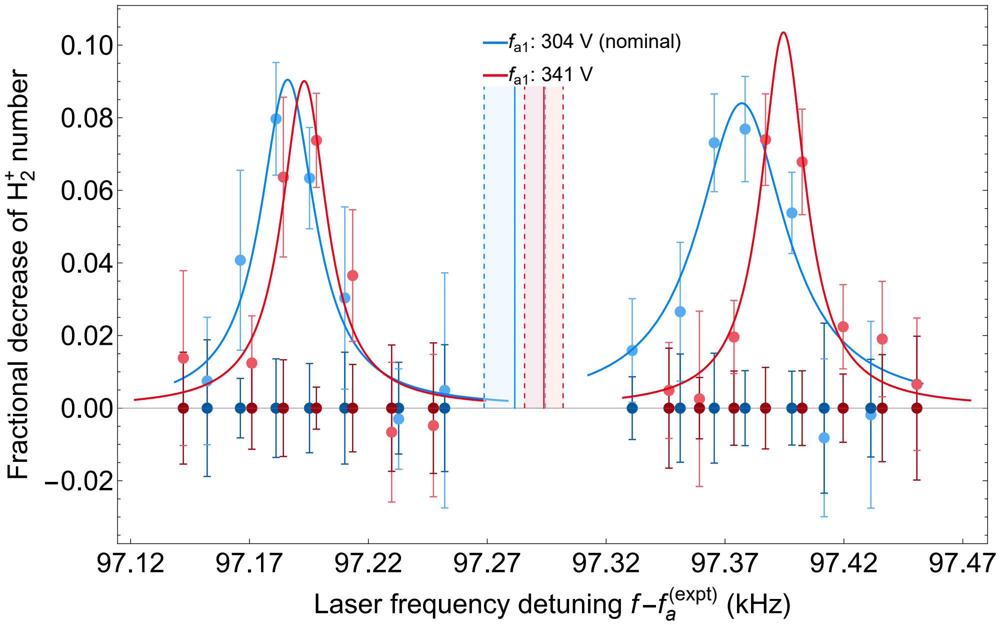

Supplementary Information Figure 1. The two AT doublets of Zeeman component f_{a1} for two different values of the trap RF amplitude. The two lines measured under nominal conditions (blue) are identical to the data shown in Fig. 2 of the main text. The laser frequency detuning is given relative to the deperturbed frequency of spin component $f_a^{(\mathrm{expt})}$. The deperturbed frequencies and their uncertainty ranges are indicated by full and dashed lines in corresponding colours. Points in brighter colour are the signal values, while the darker ones are background. The coloured curves are guides to the eye.

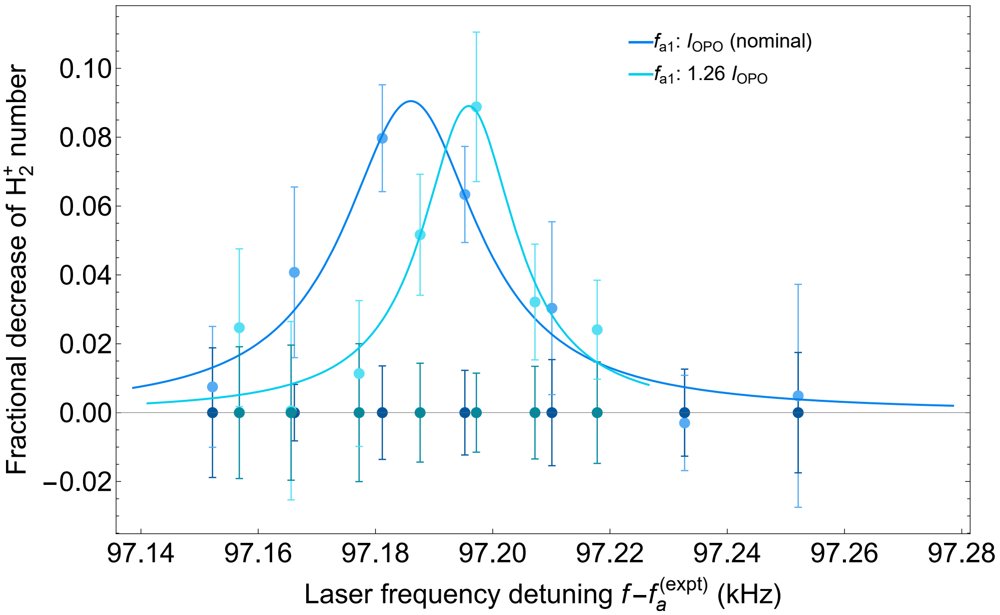

Supplementary Information Figure 2. The Zeeman component f_{a1} for two different values of the intensity of the spectroscopy wave (wavelength 2.4 μm). The line measured under nominal condition is identical to the lower component of the AT doublet shown in Fig. 2 of the main text. The laser frequency detuning is given relative to the deperturbed frequency of spin component f_a. Points in brighter colour are the signal values, while the darker ones are background. The coloured curves are guides to the eye.

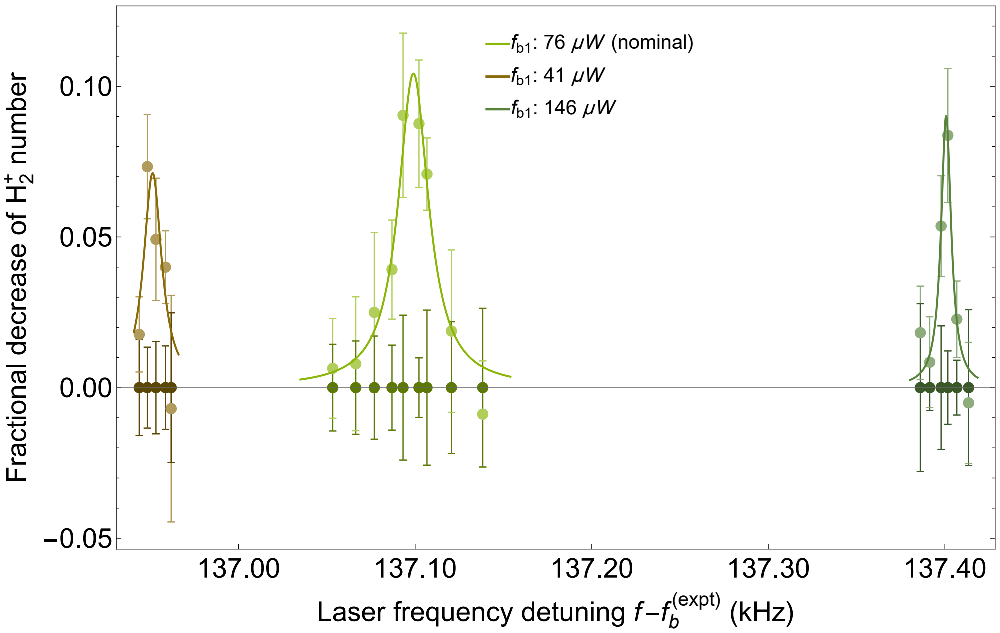

Supplementary Information Figure 3. The Zeeman component f_{b1} for three different values of the power P_{313} of the 313 nm wave. In this case, a change in power is proportional to a change in intensity, since the beam size was kept constant. The line measured under nominal condition is identical to the data shown in Fig. 3 of the main text. For each intensity setting, only one line of the AT doublet was measured and we did not identify which of the two lines was observed. However, it is probable that the three displayed lines are the same component of the AT doublet, since the observed dependence on the 313 nm wave power agrees well with the linear scaling predicted for the a.c. Stark shift of the transition. The laser frequency detuning is given relative to the deperturbed frequency of spin component f_b. Points in brighter colour are the signal values, while the darker ones are background. The coloured curves are guides to the eye. We attribute the different linewidths of the observed lines in part to a change in the reference laser of the optical frequency comb that occured inbetween the measurements (see Methods, sec. 'Details of the experiment').

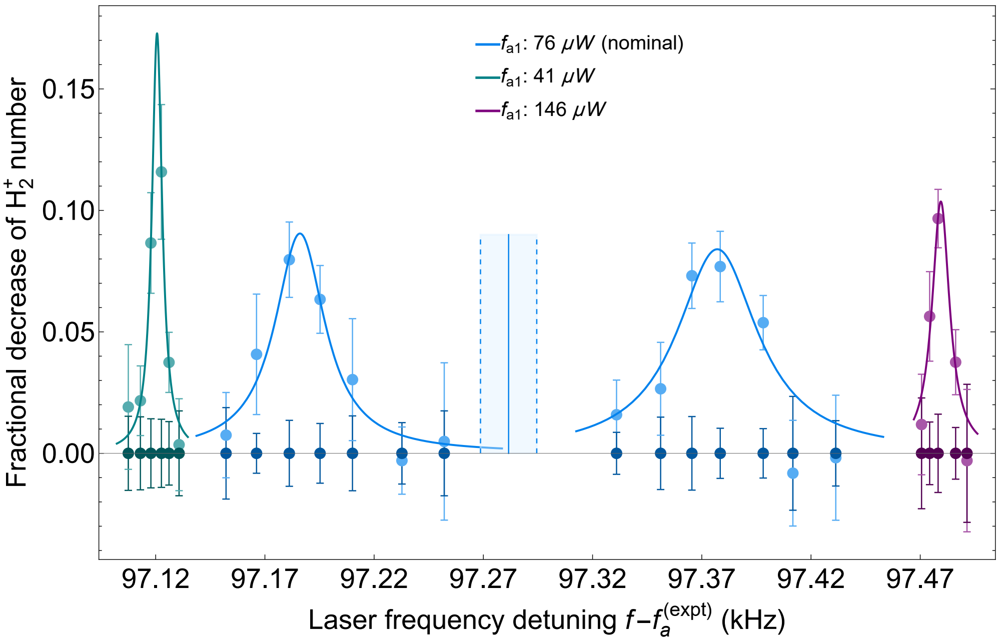

Supplementary Information Figure 4. Zeeman component f_{a1} for three different values of the power P_{313} of the 313 nm wave. In this case, a change in power is proportional to a change in intensity, since the beam size was kept constant. The AT doublet measured under nominal conditions (blue) is identical to the data shown in Fig. 2 of the main text. The laser frequency detuning is given relative to the deperturbed frequency of the spin component, f_a. Points in brighter colour are the signal values, while the darker ones are background. The coloured curves are guides to the eye. We attribute the different linewidths of the observed lines in part to a change in the reference laser of the optical frequency comb (see Methods, sec. 'Details of the experiment').

B. Polarizability ratios

effect	$r_{\text{effect,a1}}$	$r_{\text{effect,b1}}$	$r_{\text{effect,b2}}$
a.c. Stark (2.4 μm)	1.000	1.080	2.197
a.c. Stark (313 nm)	1.000	1.052	1.784
RF trap shift	1.000	1.080	2.203

Supplementary Information Table 1. Polarizability ratios $r_{\text{effect},i}$ for the three studied Zeeman components. The ratios are normalized to the polarizability of Zeeman component f_{a1}. The values are distinct for each effect, since the polarizabilities depend on the wave frequency. The ratios are derived following Methods sec. 'Evaluation of deperturbed values $f^{(\text{expt})}_{\text{spin-avg}}$ and $c^{(\text{expt})}_{\text{e}}$'. In the last row, the values of the static polarizabilities were used, since the RF frequency is small.

C. Approach (II) for spin-averaged frequency and spin-rotation coefficient

Here, we attempt to incorporate the knowledge of the discrete nature of the AT shift and to identify the unknown AT shift signs. For this purpose, we assume a particular sign $\sigma^{(j)} = \pm 1$ for each line j and set $\delta f_{\rm AT}^{(j)} = \sigma^{(j)} \Delta f_{\rm AT}^{(j)}/2$. $\Delta f_{\rm AT}^{(j)}$ has an uncertainty stemming from $\Delta f_{{\rm AT,nom},a1}^{(j)}$, and we introduce an additional uncertainty $u_{\rm AT}$ (independent of j) in order to obtain agreement between the fitted model and the data, as follows. Evaluating the LSA with this input, the uncertainties of both $f_{\rm spin\text{-}avg}^{\rm (expt)}$ and $c_{\rm e}^{\rm (expt)}$ are reduced by approximately a factor of 5 compared to the approach (I). Since seven signs $\sigma^{(j=1...7)}$ are unknown, there are $2^7 = 128$ possible combinations to consider. To determine the likelihood of particular combinations we can make use of the frequency ratios $\mathcal{R}_{5,2'}$ and $\mathcal{R}_{1,2'}$ discussed in the main text, as well as the reduced chi-squared parameter χ^2_ν resulting from the LSA. We may assume that not only the present but also our own previous experiments on HD^+ and its theory are correct at the level of the presented uncertainties. We then do not expect a substantial disagreement between the experimental and theoretical ratios. And secondly, we assume that the reduced chi-squared parameter shows a consistency between the model and the data. Our criterion for absence of disagreement in both cases is a deviation smaller than or equal to 3 sigma. For the ratios this is evaluated through their uncertainties, while for the reduced chi-squared parameter we rely on the canonical bound $\chi^2_\nu \leq \chi^2_{\nu,\rm max} = 2.9$ for the present number of degrees of freedom. If the uncertainty $u_{\rm AT} = 0$, the minimum χ^2_ν for all 128 combinations is 4.1, indicating an underestimation of the experimental errors. A total of 12 combinations have a χ^2_ν similar to this value, while the next larger χ^2_ν is 12.5, showing a clear preference of the model for these combinations. We therefore increase the uncertainty $u_{\rm AT}$ to reduce χ^2_ν until the above bound is fulfilled for the 12 combinations. We obtain χ^2_ν values in the range of 2.8 to 2.9 for $u_{\rm AT} \simeq 11\,{\rm Hz}$. Turning to the frequency ratios, only six of the 12 combinations show no disagreement regarding $\mathcal{R}_{5,2'}$, $\mathcal{R}_{1,2'}$. From these six cases we derive values for $f_{\rm spin\text{-}avg,II}^{\rm (expt)}$ and $c_{\rm e,II}^{\rm (expt)}$. We conservatively estimate them as the mid-values of the intervals containing all individual values with their uncertainties. The uncertainties are taken as equal to half the widths of the intervals. We obtain $f_{\rm spin\text{-}avg,\,II}^{\rm (expt)} = 124\,487\,032\,442.59(67)$ kHz and $c_{\rm e,\,II}^{\rm (expt)} = 34\,730.176(69)$ kHz. In summary, these uncertainties are smaller by approximately a factor 1.4 compared to approach (I).

D. Least-squares adjustment (LSA)

We perform the LSAs following ref. [1]. The basic algorithm is described in Appendix E of ref. [2]. The number of input data is n, and this input can be experimental data, theoretical data, or previously measured constants. This data can have correlations. The number of adjusted constants is M, where this output are fundamental constants and other parameters. n, M are given by

$$\begin{aligned} n &= 2\mathcal{N}(\text{MHI input}) + \mathcal{N}(\text{mass ratio input and other input} : R_{\infty,18}, r_{p,18}, r_{d,18}) + \\ &\quad \mathcal{N}(\text{other input}:\ \text{H}, \text{H - D}) + \mathcal{N}(2^{\text{nd}}\ \text{H - D datum}) \quad , \\ M &= \mathcal{N}(\text{MHI input}) + \mathcal{N}(\text{other input}:\ \text{H}, \text{H - D}) + \mathcal{N}(\text{fund. const.}) \ , \end{aligned}$$

where $\mathcal{N}(Y)$ denotes the number of items of type Y.

(i) The M adjusted constants include, depending on the particular LSA, the fundamental constants μ_{pd}/m_e, m_p/m_e, R_∞, r_p, r_d or a subset thereof. The value of m_d/m_p is computed from the adjusted μ_{pd}/m_e and m_p/m_e using the definition $\mu_{pd}/m_e = m_p m_d/m_e(m_p + m_d)$. Further adjusted "constants" are the theoretical additive corrections for the MHI, $\delta f^{(\text{theor})}(\text{HD}^+)$ and/or $\delta f^{(\text{theor})}(\text{H}_2^+)$, the so-called missing contributions. The number of adjusted MHI $\delta f^{(\text{theor})}$ is equal to the number of frequencies listed in the column "MHI input".

(ii) Every MHI input data consists of the experimental frequency $f_k^{(\text{exp})}$, the theoretical frequency $f_k^{(\text{theor})}([\mu_{pd}/m_e]_{18}, [m_p/m_e]_{18}, R_{\infty,18}, r_{p,18}, r_{d,18})$, evaluated for the CODATA 2018 fundamental constants (fiducial value), and an associated theoretical additive correction δf_k. The latter is nominally zero but has nonzero uncertainty. The δf_k of different MHI and different transitions are correlated, the correlation coefficients being given in [1].

(iii) In those LSAs where "H, H - D" is listed under "other input", "H" refers to two hydrogen 1s - 2s transition measurements (items A6, A7 in table X of ref. [3]) and "H - D" refers to one hydrogen-deuterium isotope shift measurement (A5 in table X). For the 1s - 2s measurements there is a corresponding single theoretical correction $\delta f(\text{H})$, described by B1, B2 in table VIII. For the isotope shift the theory correction $\delta f(\text{H - D})$ stems from items B1, B2, B17, B18 in table VIII. Correlation coefficients between the corrections δf are given in table IX.

Input data is analogous to the MHI input data, see (v) below. The additional adjusted

constants are $\delta f^{(\text{theor})}(\text{H})$ and $\delta f^{(\text{theor})}(\text{H - D})$. Analogous constants are considered in the CODATA adjustments, see Tab. XI in ref. [3].

(iv) In the column "Other input", we have used the short-hand notations $R_{\infty,18}$, $r_{p,18}$, $r_{d,18}$, to indicate the values of the CODATA 2018 compilation. The correlations between these values are taken into account.

(v) For each spectroscopy input datum k (from MHI, H and H - D), the observational equations are $f_k^{(\text{exp})} \doteq f_k^{(\text{theor})}(\mu_{pd}/m_e, m_p/m_e, R_\infty, r_p, r_p) + \delta f_k^{(\text{theor})}$, $\delta f_k \doteq \delta f_k^{(\text{theor})}$. If the CODATA 2018 fundamental constants are used as input instead of hydrogen spectroscopy data, then the corresponding equations are: $R_{\infty,18} \doteq R_\infty$, $r_{p18} \doteq r_p$, $r_{d,18} \doteq r_d$.

(vi) If the deuteron-proton mass ratio is an input, we include the observational equation $[m_d/m_p]_{\text{FM21}} \doteq ((m_p/m_e)/(\mu_{pd}/m_e) - 1)^{-1}$.

The observational equations are linearized around the fiducial values of the fundamental constants, i.e. the CODATA 2018 values.

In LSA 1, the constants R_∞, r_p, r_d are effectively not adjusted.

E. Autler-Townes splitting of an overtone transition of HD^+

Additional insight in the Autler-Townes (AT) splitting under coupling to a continuum is obtained from an experiment on the heteronuclear molecule HD^+. Since spectroscopy of HD^+ is much simpler to perform than on H_2^+ and yields a much higher data rate, also the AT effect is much more easily studied. In the experiment, we performed vibrational spectroscopy of the overtone transition $(v = 0, N = 0) \to (v' = 5, N' = 1)$ in the presence of a dissociation laser wave. High-accuracy spectroscopy of this transition was reported earlier, where care was taken to *suppress* the AT splitting [4].

The excitation process is a simple version of resonance-enhanced multiphoton dissociation (REMPD), in which the first (target) transition is the overtone transition driven by a laser of wavelength 1.15 μm, and the second transition (laser at 266 nm) is to a non-binding excited electronic state. SI Figure 5 shows a subset of HD^+ energy levels and these two transitions. If the target transition actually takes place, this results in a relevant probability for HD^+ dissociation.

As in the experiment in the main text, we perform repetitions of molecular ion (here, HD^+) ensemble preparation followed by REMPD. This results in a reduction of number of remaining trapped HD^+ ions. A signal proportional to this number is obtained by a resonant electric excitation of the transverse secular oscillation of the remaining HD^+ ions and observation of increased laser-induced beryllium fluorescence. We use two mid-infrared lasers (2.7 μm, 5.5 μm) to increase the fraction of HD^+ ions that are in the ground rovibrational state $(v = 0, N = 0)$. These laser waves are applied initially and are then blocked during the REMPD process.

The target transition was driven by a spectroscopy laser that was phase-locked to an optically stabilized femtosecond frequency comb. As a result, the spectroscopy laser exhibited a linewidth of less than 100 Hz. The optical frequency of the spectroscopy laser was measured with respect to a hydrogen maser that was referenced to atomic time. This permitted determining the spectroscopy laser frequency with 1×10^{-12} uncertainty.

Spectroscopy was performed on one hyperfine component of the rovibrational transition, the $(G_1 = 1, G_2 = 2, F = 2) \to (G_1' = 1, G_2' = 2, F' = 1)$ hyperfine line. Here, F is the total angular momentum of the molecule, including the rotational angulear momentum, G_1 is the total spin of the proton-electron pair, G_2 is the total particle spin, including also the

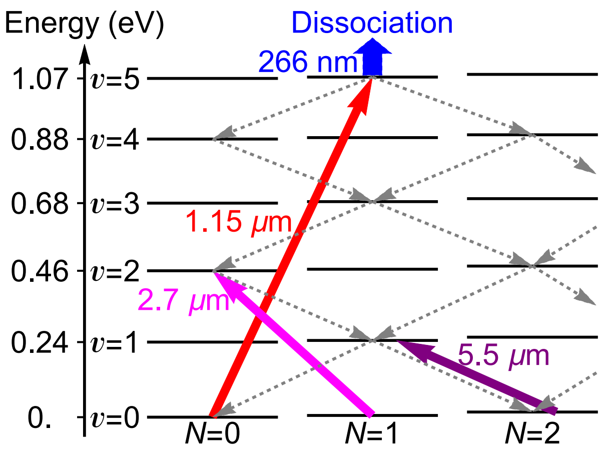

Supplementary Information Figure 5. Partial rovibrational energy diagram of HD^+ in the ground electronic state (to scale) and transitions that are driven by lasers. Initially, the magenta and purple transitions are driven. Subsequently the red and blue transitions are driven. When they are driven simultaneously an AT splitting results. The dashed arrows indicate the dominant spontaneous decay paths. v is the vibrational quantum number and N is the rotational quantum number.

deuteron. The magnetic field was set to a small value, $B = 0.0 \pm 0.2\,\mu$T. The power of the spectroscopy laser was kept constant. Three cases were studied: (i) dissociation laser off during spectroscopy laser irradiation, on afterwards (power $P_{266} = 30\,$mW), (ii) spectroscopy laser and dissociation laser on simultaneously, $P_{266} = 30\,$mW; (iii) identical to case (ii), but with a reduced dissociation laser intensity $P_{266} = 12\,$mW.

Cases (ii) and (iii) yield the data shown as blue and green data points in SI Fig. 6, respectively. These demonstrate that an AT doublet occurs if the two REMPD lasers (1.15 μm, 266 nm) are on simultaneously. Instead, case (i), the red data points, was obtained using "stepwise" REMPD where the 1.15 μm and 266 nm laser beams were unblocked in interleaved fashion. No doublet is observed. This is, of course, the preferred case for high-accuracy spectroscopy.

The spectral lines have full width at half-maximum (FWHM) of approximately 25 kHz. These relatively large linewidths may be explained by a residual magnetic field that causes a moderate splitting of the Zeeman multiplet and by power broadening induced by the spectroscopy laser on each Zeeman component, leading to spectral overlap of the components. In contrast, in our previous work [4], by applying larger magnetic fields, oriented along

the spectroscopy wave vector, we removed the degeneracy and observed individual Zeeman components with FWHM as low as 350 Hz.

The AT splitting has previously been observed for molecules, in connection with ionization [5, 6]. According to theory, the AT splitting depends on the square-root of the coupling laser's intensity. In our study the coupling laser is the 266 nm laser. SI Figure 6 shows that the splitting increases with higher laser power, and the increase is consistent with a square-root dependence on power. Since the beam diameter of the 266 nm wave was kept constant, this implies a square-root dependence on intensity. The observed splitting is quantified as 19(2) kHz/(mW)$^{1/2}\times\sqrt{P_{266}}$. The line strength for (iii) is higher than for (ii) because the higher power increases the dissociation efficiency. The line strength in case (i) is reduced because here, spontaneous decay from the upper rovibrational state is more important than for cases (ii), (iii).

Note that the power P_{266} in this study was approximately 3 orders larger than P_{313} in the H_2^+ experiment described in the main text; compare SI Fig.s 4 and 6.

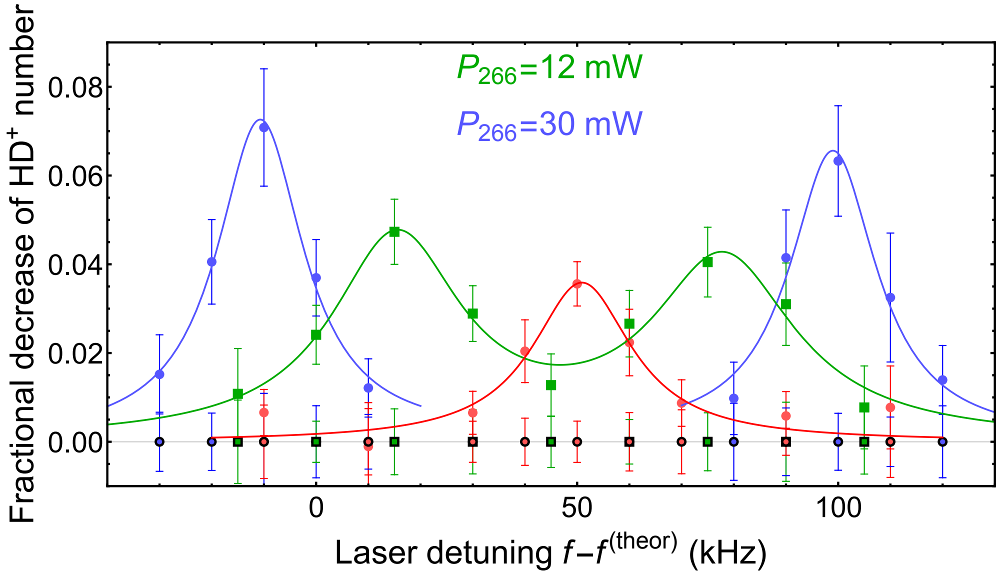

Supplementary Information Figure 6. One-photon absorption spectra of the $F = 2 \to F' = 1$ hyperfine component of the fourth vibrational overtone of HD^+ for different intensity of the simultaneously applied dissociating wave (266 nm). The lines contain unresolved Zeeman components. Red: case (i), spectroscopy laser and 266 nm dissociation laser are *not* irradiated simultaneously, but in interleaved fashion, suppressing the AT splitting. Blue and green: cases (ii), (iii), where the two lasers are irradiated simultaneously. AT doublets are clearly observed. The power of the 266 nm laser is indicated. The colored points and the colored points outlined in black indicate the REMPD-induced signal and the background loss, respectively. At each frequency the background loss was subtracted from both the colored and outlined colored data points. Lines are Lorentzian functions and are guides to the eyes. Error bars represent standard errors of the means. The measurements were performed at near zero magnetic field. f is the measured laser frequency. $f^{(\mathrm{theor})}$ is the sum of the *ab initio* computed spin-averaged transition frequency $f^{(\mathrm{theor})}_{\mathrm{spin-avg}}$ and the hyperfine frequency shift $f^{(\mathrm{theor})}_{\mathrm{spin}}$, reported in [4]. The offset of approximately 50 kHz is the recoil shift.

F. Modeling the lineshape in spectroscopy of a string of sympathetically cooled ions in a 3D cluster

While the relationship between linewidth of a spectroscopic transition and temperature of the ensemble is well-known for a gas of free particles, the answer is not obvious when the particles are confined in a trap and are interacting. The interaction becomes a key effect when the particles are so cold that they form a cluster. It would be ideal to have an accurate theory that relates a measurable quantity, the spectroscopic linewidth, to the thermodynamic quantity "temperature", which is not directly measurable. (It is not relevant for the sake of the discussion that in the present experiment the measurement of the linewidth is not easy, since it is performed on non-fluorescing molecular ions.) Currently a specific theory is not available, but simulations can provide insight.

In our work [7] we presented a model for explaining the occurrence of narrow spectral lines of sympathetically cooled (SC) ions, based on simulations. That model is a classical one; it considers the spectral intensity of the electric field $E_{\rm rad}(x_i(t))$ of the spectroscopy wave as experienced by the SC ions in the course of their motion. This motion is described classically by the coordinate $x_i(t)$ (i refers to a particular SC ion) and determined through a solution of Newton's equation. Here we follow the same model.

1. Generalities

Wittke and Dicke [8], in one of the first experimental reports of Dicke narrowing, proposed the classical description of the radiation process of moving particles. They considered an atom that starts to emit a classical wave $E_{\rm em}(t)$ at time $t = 0$, and moves along the x axis with coordinate $x(t)$,

$$\begin{aligned} E_{\rm em}(t) &= Ae^{i\omega_0 t - ikx(t)},\ t > 0, \\ &= 0,\ t < 0\,. \end{aligned}$$

A complex ω_0 is used to describe radiative decay of the atom. The real part corresponds to the mean atomic transition frequency, and $k = \mathrm{Re}(\omega_0)/c$. The quantity of primary interest is the spectrum of the radiation propagated in x direction, which is the magnitude squared

of the Fourier transform,

$$S_{\rm em}(\omega) = |H_{\rm em}(\omega)|^2 = \left| \frac{1}{\sqrt{2\pi}} \int_0^\infty e^{-i\omega t} E_{\rm em}(t) dt \right|^2 .$$

If an ensemble is probed experimentally, the ensemble average of $S_{\rm em}(\omega)$ should be computed. Note that Planck's constant appears nowhere. k is the wavenumber, not the photon momentum. The recoil effect is not described in this theory. The approach above describes Dicke line narrowing, a classical effect. The distinction between classical and quantum effects in the lineshape theory has been emphasized by Rautian [9].

In ref. [7] we have taken this approach and applied it to a trapped ion ensemble. We considered absorption of a laser or microwave wave propagating along the x direction. Conceptually, we simplified the treatment by computing only the spectrum of the incoming radiation as perceived by the moving atom

$$S_{\rm rad}(\omega) = |H(\omega)|^2 = \left| \frac{1}{\sqrt{2\pi}} \Delta t^{-1} \int_0^{\Delta t} e^{-i\omega t} E_{\rm rad}(t) dt \right|^2 , \qquad (1)$$

$$E_{\rm rad}(t) = E_0 e^{i\omega_L t - ik_L x(t)}, \text{ all } t,$$

where now ω_L is the frequency of the radiation source in the laboratory frame and Δt is the duration of the computed trajectory of a particular ion. In the linear regime (small E_0), the atomic absorption spectrum is proportional to $S_{\rm rad}$.

Although the finite Δt arises from the limitations of computing effort, it mimicks the finite interaction time of the atom with the wave. In a more general way, we might introduce a windowing function $\theta_T(t)$ of duration T that defines the interaction time, which is determined either by the experimental apparatus, experimental conditions, or because of phase-changing collisions or spontaneous emission of the atom occur. This can be recast by writing the integral as

$$S_{\rm rad}(\omega) = \left| \frac{1}{\sqrt{2\pi}} T^{-1} \int_0^T e^{-i\omega t} E_{\rm rad}(t) dt \right|^2 .$$

Additonally, an average over a distribution of values T would be taken, where the mean of the distribution would be proportional to the inverse of the atom's homogeneous linewidth. In practice, this could not be implemented in the present work, since the time duration of the available numerical trajectories $x(t)$ was only a few milliseconds, yielding an intrinsic Fourier linewidth of the spectrum larger than the experimentally observed homogenous linewidth.

2. Simulations

In the present approach, we solve the classical dynamics of a system of interacting particles and obtain the trajectories $\mathbf{r}_i$ of all particles. We then compute the lineshape for absorption of photons of a given wave vector $\mathbf{k}_L$ according to Eq. (1), $|\mathbf{k}_L| = k_L$. This vector can easily be modified in the calculation, and so it is possible to obtain the lineshape corresponding to the two most important cases: wave propagation along the symmetry axis of the trap, and perpendicular to it (the x direction). Here we focus on this latter case. The lineshape is computed for each SC ion. Subsequently, the lineshapes are averaged over all SC ions.

The classical dynamics of a multi-species ion cluster can be investigated using molecular dynamics (MD) simulations, i.e. the solution of the coupled Newton's equations for all ions in the trap, under the influence of the trap potential and the inter-particle Coulomb forces. Following the early work on sympathetically cooled particles [10–12], this approach has in the mean time been extensively applied in the ion trapping community.

In our simulations, we have used the software developed earlier [12]. The parameters of the simulation are chosen to be similar to the ones that hold in the experiment. The linear ion trap that we simulate is one in which the radial confinement is much stronger than the axial confinement. Thus, the single-ion axial frequency is $f_{\mathrm{ax,Be^+}} = 71.4\,\mathrm{kHz}$, and the radial secular frequency is $f_{\mathrm{rad,Be^+}} = 271\,\mathrm{kHz}$ for the laser-cooled ion. The axial frequency scales with ion mass M as $M^{-1/2}$, the radial frequency as M^{-1}. Thus, for H_2^+ the values are $\sqrt{9/2}$ and 9/2 times as large.

We consider a two-species ensemble with $N_{\mathrm{sc}} = 50$ H_2^+ ions and 500 beryllium ions. The H_2^+ subensemble equilibrium configuration is a string, where the ion spacing is not constant along the string.

For practical reasons, the simulated time interval was limited to 5 ms. We find that approximately 1.25 ms elapse before the mean kinetic energy of the each subspecies remains approximately constant in time. Thus, we use only the final 3.75 ms-long interval of data.

The lineshapes, averaged over all H_2^+ ions, are presented in SI Fig. 7 for a set of spectroscopy wavelengths and two radial temperatures, $T = 3.5\,\mathrm{mK}$, and $5.1\,\mathrm{mK}$. Radial temperature is defined as mean kinetic energy of radial motion times $2\,k_{\mathrm{B}}$. The lower temperature is the value we estimate to be the case for our experimental ensemble, as deduced from the Doppler width observed in axial spectroscopy on H_2^+ molecules [13]. A Dicke peak on top

of a Doppler background is found. The strength of the Dicke peak relative to the Doppler line is strongly dependent on the temperature and on the wavelength λ. For the wavelength employed in the present experimental study, the modeling yields a peak that is far stronger than the background. A simple analytical model gives a Dicke peak strength that is exponentially dependent on $-T/K_{\rm eff}\lambda^2$. Here, $K_{\rm eff}$ is the force constant of the effective binding potential for each SC ion assumed in the model. The root mean square deviation of the H_2^+ ions from the trap axis along the transverse x direction, $(N_{\rm sc}^{-1}\Sigma_i\langle x_i^2\rangle)^{1/2}$, amounts to 0.55 μm and 0.65 μm for the lower and higher temperature, respectively. Here, $\langle\ldots\rangle$ denotes the time average.

3. The recoil

We have shown above that the narrow spectral feature is a result of classical physics - a result known since Dicke's work. It is not a quantum feature. Eq. (1) does not include the effect of recoil. However, for the case of a gas of non-interacting particles, one can compute the lineshape quantum-mechanically [14]. It is a convolution of a Gaussian centered at the atomic transition frequency, shifted by recoil, and a Lorentzian due to the natural spectral broadening. In the limit of small Lorentzian broadening, to very good approximation a shifted Gaussian is obtained. The result of incorporating quantum effects is thus to shift the whole Gaussian line, and this is the case at any temperature, even at high temperatures. As this Gaussian line is also found in a purely classical analysis that per definition assumes high temperature and has no recoil, we deduce that the quantum correction to a classical spectrum is a shift of the whole spectrum. It is plausible that the spectrum derived above for a classical, trapped ion system is also shifted as a whole, that is, both the narrow feature as well as the underlying Gaussian feature are shifted equally. This would be true as long as the system is in the classical regime.

We emphasize that our trapped mixed ion ensemble is in the classical regime, as its thermal energy (see above) is substantially above even the highest phonon energy of the system, given by the transverse secular frequency. Moreover, this frequency does not by itself characterize the ensemble. We performed a numerical calculation of the modes for the equilibrium state of a two-species cluster at zero temperature. Due to computational complexity, the size of the cluster was substantially smaller than in the experiment: 30 HD^+

ions and 270 Be^+ ions, with the HD^+ ions in a string configuration. Many modes have frequencies below 50 kHz, far less than the HD^+ single-particle transverse secular frequency, and some have frequencies smaller than the recoil frequency (17 kHz). Expressed in simple terms, the ions are not tightly, but softly bound in the cluster. The mode spectrum at the lowest frequencies depends sensitively on the number of ions.

Recently, a simulation of high-resolution spectroscopy of an intermediate-size single-species 3D cluster containing 1000 atomic ions (Sn^{2+}) has been reported [15]. The assumed trap parameters were chosen to yield an anisotropic transverse quasipotential and a high axial secular frequency. For the assumed particle number a strongly elongated 3D cluster with elliptical cross section results. The simulations were performed for low ion temperature, 0.1 mK, to be achieved using Doppler cooling by three noncolinear cooling beams. The spectrum of an optical transition excited by an axially propagating spectroscopy wave was computed, assuming an irradiation time of 1 ms. It shows a resolved carrier, free of recoil, and sidebands from cluster modes. Noise effects were not taken into account.

The parameters of this model system and its simulation are substantially different from those realized in our experiment, which provides only an axial Doppler cooling beam, resulting in substantially higher temperature. Furthermore, our ensemble is trapped in an isotropic transverse quasi potential. The higher temperature implies stronger thermal fluctuations causing stronger random variations of the cluster configuration. The cluster is also affected by technical noise both on the cooling laser intensity and on the trap electrodes voltages. Additionally, a significant effect arises from our use of a destructive detection scheme: during the 20-s-long spectroscopy phase, approximately 10–15% of the trapped MHIs leave the trap following dissociation. Hence, the mode frequencies are not stable in time, specifically they vary over the timescale of excitation of the spectroscopy transition. Our non-ideal conditions differ subtantially from those assumed in deriving theoretically the existence of a recoilless carrier transition (Mössbauer effect) - specifically a time-independent mode spectrum - and furnish an argument for the appearance of a recoil shift in our system.

We expect that in the near future the same transition will be measured on a single trapped molecular ion or in a quantum-logic-spectroscopy two-ion setting, where control over the ion(s) can be much stronger. The comparison of those transition frequencies with the one of the present work will allow determining the recoil shift directly.

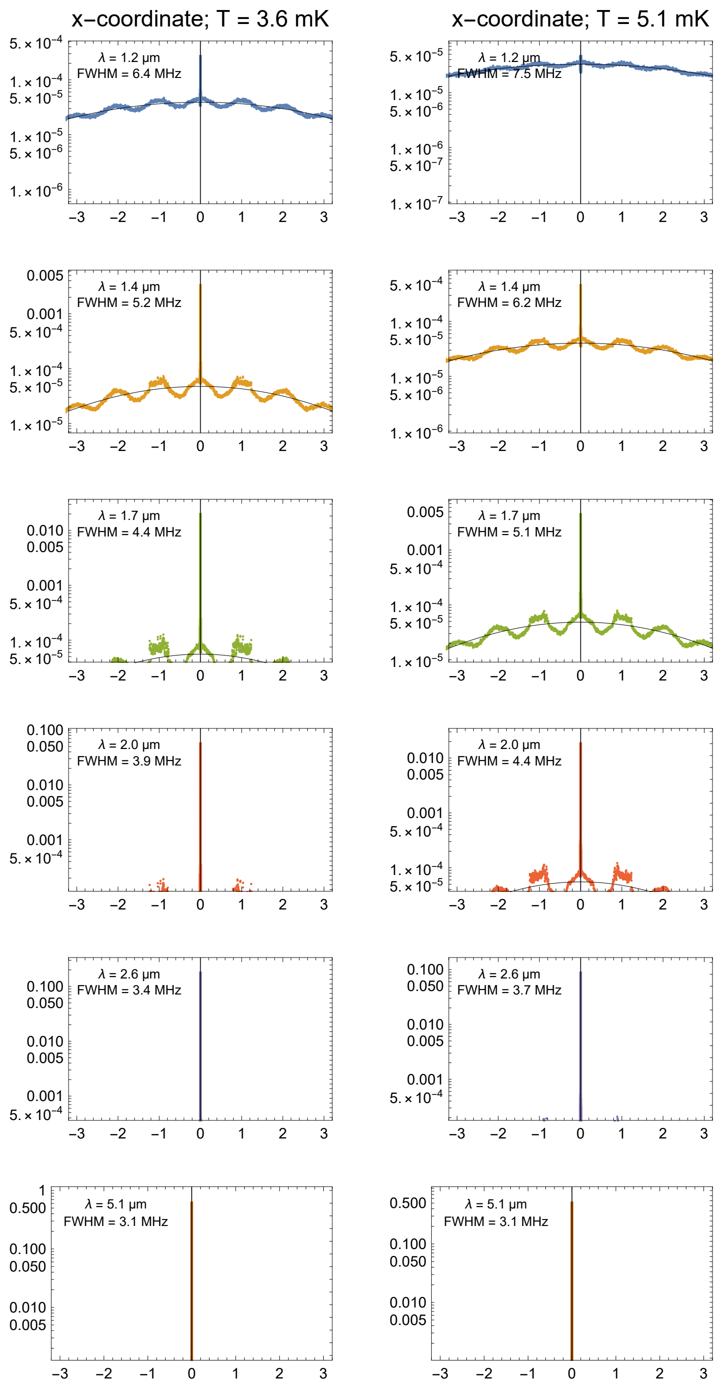

Supplementary Information Figure 7. Simulated absorption lineshapes for wave propagation along the x direction (transverse to the trap axis) for two different temperatures of the x motion and different spectroscopy wavelengths λ. The horizonal axis is the laser detuning from resonance, in MHz. The vertical axis shows $S_{\rm rad}$ in arbitrary units. The full-width at half-maximum (FWHM) of the spectrally broad (Doppler) background is given in the plot legends. The black line in some of the panels shows a Gaussian fit to the background.

[1] S. Schiller, J.-P. Karr, Prospects for the determination of fundamental constants with beyond-state-of-the-art uncertainty using molecular hydrogen ion spectroscopy, Phys. Rev. A 109 (2024) 042825. doi:10.1103/PhysRevA.109.042825.

[2] P. J. Mohr, B. N. Taylor, CODATA recommended values of the fundamental physical constants: 1998, Rev. Mod. Phys. 72 (2000) 351–495. doi:10.1103/RevModPhys.72.351.

[3] E. Tiesinga, P. J. Mohr, D. B. Newell, B. N. Taylor, CODATA recommended values of the fundamental physical constants: 2018, Rev. Mod. Phys. 93 (2021) 025010. doi:10.1103/RevModPhys.93.025010.

[4] S. Alighanbari, I. V. Kortunov, G. S. Giri, S. Schiller, Test of charged baryon interaction with high-resolution vibrational spectroscopy of molecular hydrogen ions, Nat. Phys. 19 (2023) 1263–1269. doi:10.1038/s41567-023-02088-2.

[5] J. Kim, J. S. Lim, H.-R. Noh, S. K. Kim, Experimental Observation of the Autler-Townes Splitting in Polyatomic Molecules, J. Phys. Chem. Lett. 11 (2020) 6791–6795. doi:10.1021/acs.jpclett.0c01918.

[6] M. A. Quesada, A. M. F. Lau, D. H. Parker, D. W. Chandler, Observation of Autler-Townes splitting in the multiphoton ionization of H_2: Measurement of vibronic transition moments between excited electronic states, Phys. Rev. A 36 (1987) 4107–4110. doi:10.1103/PhysRevA.36.4107.

[7] S. Alighanbari, M. G. Hansen, V. I. Korobov, S. Schiller, Rotational spectroscopy of cold and trapped molecular ions in the Lamb-Dicke regime, Nat. Phys. 14 (2018) 555. doi:10.1038/s41567-018-0074-3.

[8] J. P. Wittke, R. H. Dicke, Redetermination of the Hyperfine Splitting in the Ground State of Atomic Hydrogen, Phys. Rev. 103 (1956) 620–631. doi:10.1103/PhysRev.103.620.

[9] S. G. Rautian, On Interpretation of the Dicke Effect, Opt. Spectrosc. 102 (2007) 315–317. doi:10.1134/S0030400X07030010.

[10] S. Schiller, C. Lämmerzahl, Molecular dynamics simulation of sympathetic crystallization of molecular ions, Phys. Rev. A 68 (2003) 053406. doi:10.1103/PhysRevA.68.053406.

[11] P. Blythe, B. Roth, U. Fröhlich, H. Wenz, S. Schiller, Production of ultracold trapped molecular hydrogen ions, Phys. Rev. Lett. 95 (2005) 183002. doi:10.1103/PhysRevLett.95.183002.

[12] C. B. Zhang, D. Offenberg, B. Roth, M. A. Wilson, S. Schiller, Molecular-dynamics simulations of cold single-species and multispecies ion ensembles in a linear Paul trap, Phys. Rev. A 76 (2007) 012719. doi:10.1103/PhysRevA.76.012719.

[13] M. R. Schenkel, S. Alighanbari, S. Schiller, Laser spectroscopy of a rovibrational transition in the molecular hydrogen ion H_2^+, Nat. Phys. 20 (2024) 383–388. doi:10.1038/s41567-023-02320-z.

[14] K. S. Singwi, A. Sjölander, Resonance Absorption of Nuclear Gamma Rays and the Dynamics of Atomic Motions, Phys. Rev. 120 (1960) 1093–1102. doi:10.1103/PhysRev.120.1093.

[15] D. R. Leibrandt, S. G. Porsev, C. Cheung, M. S. Safronova, Prospects of a thousand-ion Sn^{2+} Coulomb-crystal clock with sub-10^{-19} inaccuracy, Nat. Commun. 15 (2024) 5663. doi:10.1038/s41467-024-49241-w.
